# Mahogunin Ring Finger 1 Is Required for Genomic Stability and Modulates the Malignant Phenotype of Melanoma Cells

**DOI:** 10.3390/cancers12102840

**Published:** 2020-10-01

**Authors:** Idoya Martínez-Vicente, Marta Abrisqueta, Cecilia Herraiz, Julia Sirés-Campos, María Castejón-Griñán, Dorothy C. Bennett, Conchi Olivares, Jose Carlos García-Borrón, Celia Jiménez-Cervantes

**Affiliations:** 1 Department of Biochemistry, Molecular Biology and Immunology, School of Medicine, University of Murcia and Instituto Murciano de Investigación Biosanitaria (IMIB), 30120 Murcia, Spain; idoyamaria.martinez@um.es (I.M.-V.); marta.ag@um.es (M.A.); ceciliahs@um.es (C.H.); juliasc@um.es (J.S.-C.); maria.castejon1@um.es (M.C.-G.); mcolisan@um.es (C.O.); gborron@um.es (J.C.G.-B.); 2 Molecular and Clinical Sciences Research Institute, St. George’s, University of London, London SW17 0RE, UK; dbennett@sgul.ac.uk

**Keywords:** Mahogunin Ring Finger 1, melanoma, melanocytes, genomic stability, DNA damage, cell cycle

## Abstract

**Simple Summary:**

Melanoma, the most aggressive skin cancer, accounts for the majority of deaths due to this disease. Therefore, identification of genes/proteins involved in melanoma genesis and/or progression is urgent. Mutations abrogating expression of Mahogunin Ring Finger 1 (MGRN1) in mice cause complex phenotypes with hyperpigmentation, and known MGRN1 interactors are important regulators of cell shape and movement. This suggests that MGRN1 may modulate the malignant phenotype of melanoma cells. Analysis of MGRN1-KO mouse melanocytes and melanoma cells showed that lack of MGRN1 leads to cell cycle defects and to a more differentiated, less aggressive phenotype, with increased adhesion to various matrices, decreased motility and high genomic instability. The higher aggressivity of MGRN1-expressing melanoma cells was confirmed in an in vivo mouse melanoma model and is consistent with higher survival of human melanoma patients expressing low levels of MGRN1. Therefore, MGRN1 appears an important determinant of the malignant phenotype of melanoma.

**Abstract:**

The mouse *mahoganoid* mutation abrogating Mahogunin Ring Finger-1 (MGRN1) E3 ubiquitin ligase expression causes hyperpigmentation, congenital heart defects and neurodegeneration. To study the pathophysiology of MGRN1 loss, we compared *Mgrn1*-knockout melanocytes with genetically matched controls and melan-md1 (*mahoganoid*) melanocytes. MGRN1 knockout induced a more differentiated and adherent phenotype, decreased motility, increased the percentage of cells in the S phase of the cell cycle and promoted genomic instability, as shown by stronger γH2AX labelling, increased burden of DNA breaks and higher abundance of aneuploid cells. Lack of MGRN1 expression decreased the ability of melanocytes to cope with DNA breaks generated by oxidizing agents or hydroxyurea-induced replicative stress, suggesting a contribution of genomic instability to the *mahoganoid* phenotype. MGRN1 knockout in B16-F10 melanoma cells also augmented pigmentation, increased cell adhesion to collagen, impaired 2D and 3D motility and caused genomic instability. Tumors formed by *Mgrn1*-KO B16-F10 cells had lower mitotic indices, fewer Ki67-positive cells and showed a trend towards smaller size. In short-term lung colonization assays *Mgrn1*-KO cells showed impaired colonization potential. Moreover, lower expression of MGRN1 is significantly associated with better survival of human melanoma patients. Therefore, MGRN1 might be an important phenotypic determinant of melanoma cells.

## 1. Introduction

Mahogunin Ring Finger-1 (MGRN1) is a C3HC4 RING-Finger motif-containing nuclear-cytoplasmic E3 ubiquitin ligase well conserved throughout the phylogenetic scale [1,2] and ubiquitously expressed in mammals [3]. In mice, it is encoded by *Mgrn1*, site of the coat color mutant allele *mahoganoid* (*Mgrn1^md^*) [3] on chromosome 16. Homozygous *mahoganoid* mutant mice lack MGRN1 expression and show darker pigmentation on an agouti or yellow background compared with *Mgrn1* wild-type animals, that is, the mutation tends to replace yellow pheomelanin with black eumelanin, likely by modulating signaling from the melanocortin receptor MC1R [2,3,4]. *Mahoganoid* mice have pleiotropic phenotypes that affect different cell types [5], suggesting that MGRN1 is important for other biological processes, in addition to the regulation of skin pigmentation. Adult homozygous animals develop progressive spongiform neurodegeneration with central nervous system (CNS) vacuolation and features of prion diseases, but without accumulation of prion proteins [2,6]. These mice also show mitochondrial dysfunction, with reduced expression and activity of electron transport chain proteins and increased oxidative stress in the CNS [7], aberrant patterning of the left-right body axis, congenital heart defects [8], abnormal cranial shape [9] and high embryonic lethality [8]. MGRN1 deficiency also causes male infertility, disruption of hormonal secretion and impaired sperm motility [10]. To date no phenotype like *mahoganoid* has been described in humans and *MGRN1* point mutations are rare (cancer.sanger.ac.uk/cosmic) [11,12]. 

The mouse and human *MGRN1* genes are orthologs with 17 exons, that yield at least four protein-coding isoforms by alternative splicing of exons 12 and 17 [4,13]. These isoforms are not functionally equivalent [4,9], since overexpression only of certain MGRN1 isoforms rescued the normal pigmentation pattern [9]. All isoforms share exons 1–11, and, therefore, harbor the RING Finger domain encoded by exon 10. This domain is the hallmark of E3 ubiquitin ligases [14], responsible for catalyzing the conjugation of ubiquitin (Ub) units to target proteins. Indeed, MGRN1 displays E3 ligase activity towards multiple protein substrates [15]. These include TSG101, a component of the endosomal sorting complex required for transport-1 (ESCRT1) [6,16,17,18,19], Mitofusin1 and GP78 which contribute to the control of mitochondrial dynamics [20,21,22,23] and α-Tubulin (α-TUB) but not β-TUB or γ-TUB [21,24]. In addition, co-immunoprecipitation experiments demonstrated the interaction of MGRN1 with NEDD4, a HECT-domain ubiquitin ligase involved in endosomal trafficking, although no evidence of MGRN1-dependent ubiquitination of NEDD4 was found [16]. Accordingly, it has been proposed that MGRN1 modulates endosomal trafficking [16,17,19], microtubule stability [24,25] and mitotic spindle orientation [24,25,26], thus potentially playing a role in cell division. MGRN1 may also target misfolded proteins by interaction with the molecular chaperone HSP70 [27] and with polyglutamine (PQ) proteins such as Huntingtin and Ataxin-3 [28], most likely to suppress PQ and misfolded proteins aggregation and toxicity [29]. Two MGRN1 isoforms contain a canonical nuclear localization signal (NLS) in exon 12. These isoforms translocate from the cytosol to the nucleus under regulated conditions not yet explored in detail [13]. MGRN1 was shown to move from the cytoplasm to the nucleus in aging neurons, to potentiate a transcriptional response to stress that improves neuronal survival [30]. MGRN1 also delays forward trafficking of the Amyloid Precursor Protein through the secretory pathway, thus inhibiting its proteolytic processing and hence the release of amyloidogenic peptides to the extracellular medium of cultured heterologous cells or hippocampal neurons [31]. In this regard, sequestration of MGRN1 in the cytosol by forced expression of cytosolically exposed forms of the prion protein partially phenocopied MGRN1 depletion, as it led to lysosomal alterations in cultured cells and animal models [32]. Accordingly, cytosolic sequestration of MGRN1 was postulated to contribute to neurodegeneration [32] but no evidence of such misslocalization of MGRN1 in normal or pathological situations has yet been provided. Overall, these data together with the neurodegeneration in mutant mice point to a positive role of nuclear MGRN1 in protection against certain stresses. Additionally, MGRN1 modulates the function of several members of the melanocortin receptor subfamily of G protein-coupled receptors (GPCRs), including MC1R, MC2R and MC4R [13,33,34,35].

*Mgrn1*-null melan-md1 mouse melanocytes derived from *Mgrn1^md-nc^/Mgrn1^md-nc^* mice are more pigmented in vitro than control *Mgrn1^+/+^* cells [36], indicating that hyperpigmentation of *md* mice is a cell-autonomous process, as previously suggested by genetic studies of mutant mice [37]. Other molecular consequences of loss of MGRN1 expression on key melanocyte processes remain poorly known. We report here the first study on the biological consequences of lack of MGRN1 on motility, cell cycle progression and genomic stability, using a panel of *Mgrn1*-null melanocytes and melanoma cells. We show that MGRN1 deficiency increases genomic instability and leads to a more differentiated phenotype with significant changes in the metastatic potential of mouse melanoma cells.

## 2. Results

### 2.1. Effects of MGRN1 Ablation on Melanocyte Morphology and Motility

To investigate the biological effects of MGRN1 deficiency, we used immortalized *Mgrn1*-null (*Mgrn1^md-nc^/Mgrn1^md-nc^*) melan-md1 (*mahoganoid*-null) mouse melanocytes [36] and CRISPR/Cas9 *Mgrn1*-KO clonal melan-a6 melanocytes, as well as genetically matched melan-a6 control cells. The strategy for the generation of *Mgrn1*-KO clones is described in Figure S1A. Clones were selected based on lack of MGRN1 expression (Figure S1B) and confirmed by *Mgrn1* gene amplification and sequencing (Figure S1C). Three independent clones were isolated for further analysis, and the absence of wildtype cells in the clonal cultures was confirmed by genomic DNA sequencing and allele-specific PCR using primers specific for the native gene (data not shown). These clones displayed early disruption of the *Mgrn1* open reading frame leading to an altered protein sequence following the first 22 amino acids and a premature truncation. Therefore, the resulting polypeptides lack the relevant functional domains in wildtype MGRN1, including the NLS and the RING motif. 

**Figure 1:**
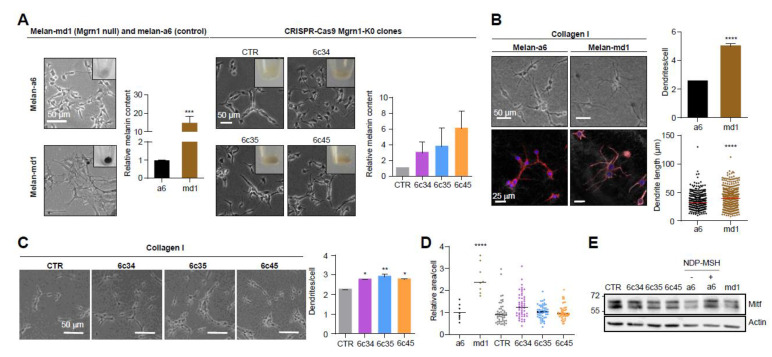
Induction of a more differentiated phenotype in mouse melanocytes lacking MGRN1 expression. (**A**) Morphology of melan-a6 and melan-md1 cells (left) and CRISPR/Cas9 *Mgrn1*-KO clones 6c34, 6c35 and 6c45 (right) in 2D cultures. The inserts in the phase-contrast images show representative images of cell pellets. Histograms correspond to quantification of melanin content normalized for melan-a6 controls in the case of melan-md1 cells (*n* = 6), and for cells transfected with a non-targeting crRNA and selected with puromycin (CTR) for CRISPR/Cas9 clones (*n* = 3). (**B**) Increased dendricity of melan-md1 cells seeded on collagen I. Phase-contrast (upper) and confocal images (lower) stained for F-actin (red) and for nuclei (Hoechst staining, blue) are shown for melan-a6 and -md1 cells. Histograms show the quantification of the number of dendrites per cell (*n* = 100 randomly selected cells) and the length of dendrites (*n* = 300) in melan-a6 and melan-md1 cells on collagen I (right). (**C**) Phase-contrast images of *Mgrn1*-KO clones on collagen I (left) and quantification of the number of dendrites per cell (right). (**D**) Relative area per cell in melan-a6 (control), melan-md1 and *Mgrn1*-KO clonal cells. Data are normalized for the control melan-a6 cells. (**E**) Lack of MITF overexpression in MGRN1-null melanocytes. A representative immunoblot for MITF in melan-a6 and *Mgrn1*-null cells is shown. As a positive control, melan-a6 cells were treated with NDP-MSH (10^−7^ M for 24 h) to ascertain induction of MITF. * indicates *p* < 0.05, **, *p* < 0.01, ***, *p* < 0.001 and ****, *p* < 0.0001.

*Mahoganoid*-null and *Mgrn1*-KO melanocytes grown on 2D cultures in plastic plates showed a more differentiated phenotype, characterized by higher melanin content and a more dendritic appearance compared to the mouse littermate control cells melan-a6 (Figure 1A). A more dendritic and heavily pigmented phenotype of melan-md1 cells has been previously reported [36].

We confirmed the switch to a more differentiated morphology in melan-md1 melanocytes seeded in 2½D collagen systems, better mimicking the dermal extracellular matrix (Figure 1B). In these culture conditions, melan-md1 cells displayed twice as many dendrites as melan-a6 controls, and the dendrites were significantly longer. Clonal *Mgrn1*-KO cells also showed significantly higher dendricity than controls, but the differences were less marked (Figure 1C). On the other hand, melan-md1 cells were significantly bigger than melan-a6 controls (Figure 1D). There was also a trend towards increased cell size for *Mgrn1*-KO clones. Finally, we inhibited MGRN1 expression in melan-a6 cells using two specific siRNAs, separately or in combination (Figure S2A,B). Depletion of MGRN1 increased dendricity (Figure S2C,D) and size (Figure S2E).

The microphthalmia-associated transcription factor (MITF) is a central regulator of melanocyte biology [38]. High levels of MITF are associated with a highly differentiated and pigmented phenotype and a low proliferation rate. Since MITF undergoes ubiquitylation leading to proteolysis, we tested the possibility that lack of MGRN1 E3 ligase might stabilize MITF, thus accounting for the differentiated phenotype of *Mgrn1*-KO cells. However, the levels of MITF were similar in *Mgrn1*-null cells compared with their controls (Figure 1E), thus ruling out MITF stabilization as responsible for the *mahoganoid* phenotype of melanocytes.

**Figure 2:**
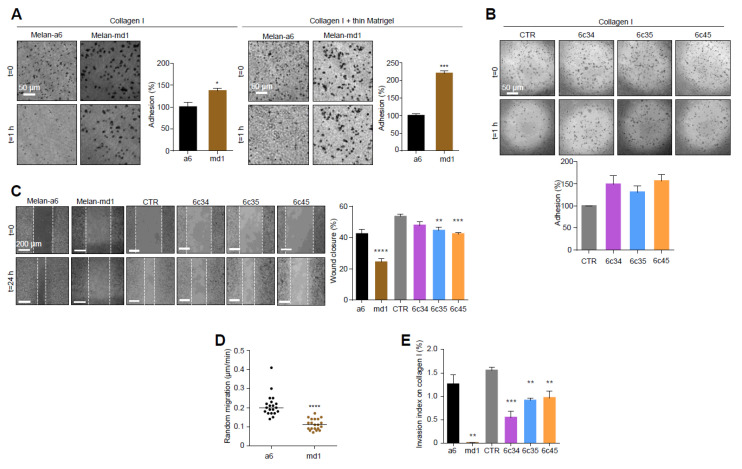
Changes in cell adhesion and motility in *Mgrn1*-null melanocytes. (**A**) Phase-contrast images of melan-a6 and melan-md1 cells adhering to collagen I (left) and to a thin layer of matrigel on top of a thick layer of collagen I (right) after 1 h, and quantification of percentage of adhesion relative to control cells. (**B**) Higher adhesion of *Mgrn1*-KO melan-a6-derived clones adhering to collagen I, quantified as in panel A. (**C**) Wound healing assay of melan-a6 and melan-md1 cells (left) and *Mgrn1*-KO clones (right). The phase contrast images of monolayers of the indicated cells show the extent of closure 24 h after a scratch. The quantification of the percentage of wound closure is shown in the bar graph. (**D**) Random migration of melan-a6 and melan-md1 cells on collagen I. (**E**) Altered invasion through collagen I after 24 h of *Mgrn1*-null melan-md1 and *Mgrn1*-KO clones relative to melan-a6 control cells. * indicates *p* < 0.05, **, *p* < 0.01, ***, *p* < 0.001 and ****, *p* < 0.0001.

In the light of the extensive crosstalk between cell shape and motility [39], we analyzed the adhesion and migration behavior of *Mgrn1*-null cells. MGRN1 ablation resulted in higher attachment to collagen I or collagen I + thin Matrigel (Figure 2A), where the adhesion of melan-md1 melanocytes was 2-fold higher relative to controls. Similar results were obtained for *Mgrn1*-KO clones (Figure 2B). Moreover, 2D cell motility measured by wound healing assays (Figure 2C) and random movements over 24 h (Figure 2D) was significantly reduced in cells lacking MGRN1. Both melan-md1 and *Mgrn1*-KO cells also showed decreased 3D invasion into a collagen I matrix with serum-enriched medium as chemoattractant (Figure 2E).

Silencing *Mgrn1* gene expression in melan-a6 cells by specific siRNAs had similar effects on 2D and 3D motility (Figure S2F,G). Therefore, *Mgrn1* knockdown led to a differentiated phenotype with increased dendricity, pigmentation and size but lower motility. This phenotypic switch occurred without a significant change in Mitf expression.

### 2.2. Effect of Mgrn1 Knockdown on Cell Cycle Progression

**Figure 3:**
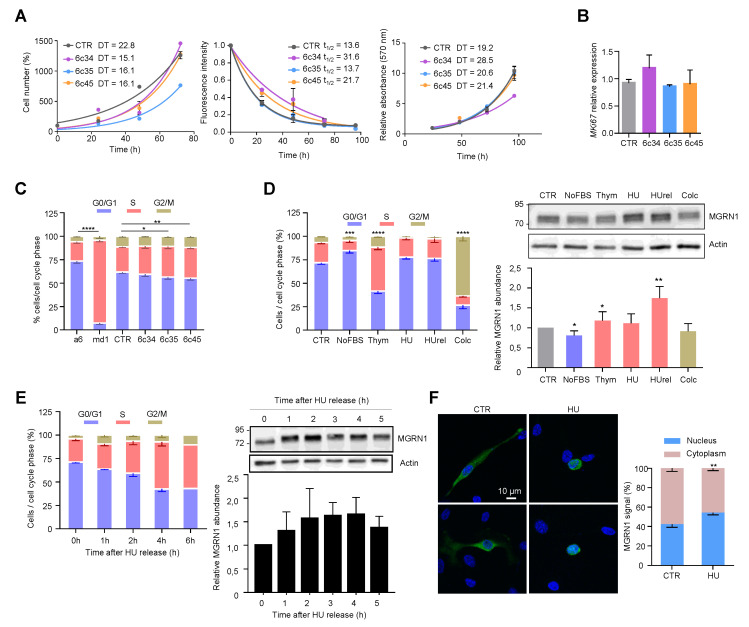
Cell cycle-dependent modulation of MGRN1 expression in mouse melanocytes. (**A**) Left: Growth curves for *Mgrn1*-KO clones in 2D cultures as determined by cell counting (represented as percentage of cell number relative to control at day 1 for a period of 4 days. Middle: Kinetics of decay of CFSE fluorescence intensity of melan-a6 and melan-md1 cells after labeling cells with an amine-reactive dye. Right: MTT assay represented as relative absorbance at 570 nm over 4 days. The corresponding doubling times (DT) or half-lives (*t*_1/2_) were calculated by non-linear regression and are shown in each graph as mean ± sem (*n* ≥ 3). (**B**) Relative expression of *Mki67* in control and *Mgrn1*-KO estimated by qPCR (*n* = 4). (**C**) Cell cycle analysis for propidium iodide-stained melan-a6 and *Mgrn1*-null cells (*n* = 6 for melan-a6 and –md1 cells, *n* = 12 for CRSPR/Cas9 *Mgrn1*-KO cells). The statistical analysis shown refers to the percentage of cells in S phase. (**D**) Left, cell cycle analysis of PI-stained melan-a6 cells synchronized by serum starvation (NoFBS), or treatments with thymidine (Thym), hydroxyurea (HU), HU followed by washoff and 1 h release in HU-free medium (HUrel) and colcemid (Colc). Statistical analysis as in panel C. Right, representative immunoblots for MGRN1 in synchronized cells. The histogram below the blot corresponds to the quantification of the intensity of the MGRN1 band, normalized to control asynchronous cell extracts (CTR). Results given as mean ± sem (*n* = 3 or higher). (**E**) Left, time-dependent changes of cell cycle distribution for cells treated with HU for 4 h, followed by washoff and release in HU-free medium for the times shown. Right, representative immunoblot for MGRN1 and quantification of the relative MGRN1 expression at the different time points after release (*n* ≥ 2). (**F**) Melan-a6 cells transfected to express the HA-labeled S(+) isoform of MGRN1 were treated for 4 h with vehicle (CTR) or with HU, as indicated. Cells were stained for nuclei (with DAPI, blue signal) and for MGRN1 (with an αHA antibody, green signal), and observed in a confocal microscope. Representative images are shown on the left. The bar graph shows the percentage of the total MGRN1 signal associated to nuclei. * indicates *p* < 0.05, **, *p* < 0.01, ***, *p* < 0.001 and ****, *p* < 0.0001.

Data on the effect of MGRN1 on cellular proliferation and cell cycle progression are still scarce and inconclusive. Previous work showed similar growth rates for melan-md1 and melan-a6 cells, suggesting that lack of MGRN1 has little impact on cell proliferation, at least on the *Cdkn2a*-deficient background of these cell lines [36]. On the other hand, a genome-wide functional screening for human cell cycle regulators performed by targeting > 95% of the protein-coding genes with siRNA found increased number of cells in S phase in MGRN1-silenced U2OS cells [40], which was not investigated further. To extend these findings, we analyzed the growth and cell cycle progression of control and CRISPR-generated *Mgrn1*-KO melanocytes. Proliferation rates of control and *Mgrn1*-KO melanocytes in 2D cultures were compared by means of three different techniques, namely the trypan blue method (Figure 3A left), measurement of the fluorescence decay of an amine-reactive dye (Figure 3A, middle) and the MTT assay (Figure 3A, right). We did not find consistent and statistically significant differences between control and *Mgrn1*-KO cells. Moreover, the expression of the proliferation marker Mki67 was similar in control compared with *Mgrn1*-KO clones (Figure 3B). siRNA-mediated depletion of MGRN1 did not cause a significant change in the proliferation rate of melan-a6 cells grown on plastic dishes (Figure S3A). In 3D cultures, the growth of spheroids derived from *Mgrn1*-KO clones or control cells was also comparable (Figure S3B).

Next, we analyzed the mitotic cell cycle of *Mgrn1*-null cells. In asynchronous cultures, cell cycle profiles suggested a massive retention of melan-md1 cells in S phase (Figure 3C). *Mgrn1*-KO clonal cells also showed a significant, but less dramatic, trend towards increased percentage of cells in S phase (Figure 3C). In summary, ablation of MGRN1 expression interfered with progression through the cell cycle, as it was associated with an increased fraction of cells in S phase, but its effect on the rate of proliferation was not significant. This phenotype was consistent with previous reports by others [36,40] and was mild in *Mgrn1*-KO cells but much more severe in melan-md1 cells. This suggested that the aberrant cell cycle profile of melan-md1 cells could be due not only to loss of MGRN1 but also to a genetic drift in culture. Accordingly, further studies on the alteration of cell cycle progression were performed exclusively using *Mgrn1*-KO clones.

**Figure 4:**
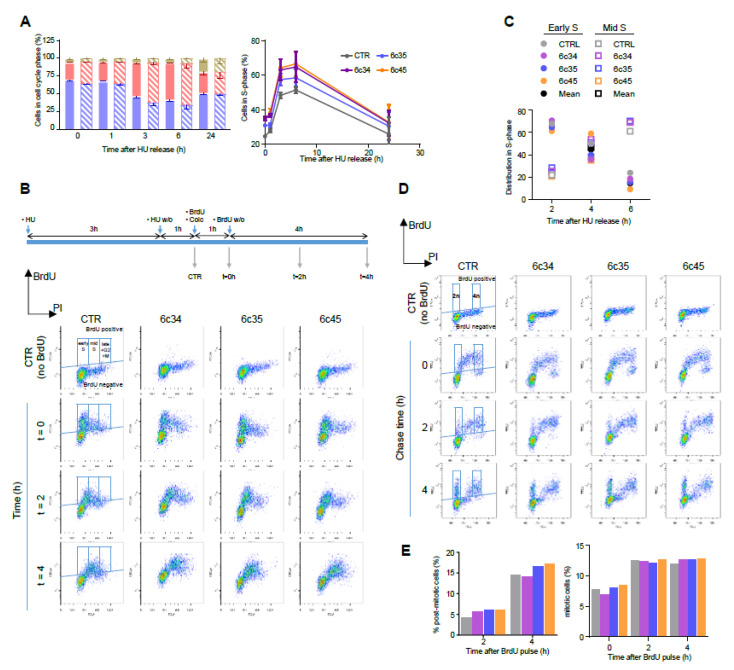
Kinetics of cell cycle progression in *Mgrn1*-KO cells. (**A**) Left, cell cycle analysis of control (solid bars) and *Mgrn1*-KO 6c35 cells (dashed bars) at different times following release from block at the G1/S interphase with HU (2 mM, 3 h). Right, kinetics of changes in the fraction of cells in S phase upon release from the HU block for control (CTR) and *Mgrn1*-KO clones. (**B**) Pulse-chase analysis of cell cycle progression upon release from a HU-block. CTR cells and *Mgrn1*-KO clones treated with HU (2 mM, 3 h) were washed-off and pulsed with BrdU (1 h) in the presence of colcemid. Cells were further incubated in colcemid-containing BrdU-free medium and analyzed for BrdU intensity and DNA content (PI stain). The upper scheme shows the experimental design. Plots of BrdU intensity vs. DNA content are shown below. Boxes indicate the gates used for further analysis. (**C**) Time-dependent changes of the distribution in early and mid S phase of CTR or *Mgrn1*-KO BrdU-labelled cells, according to gating scheme in panel B. (**D**) Plots of BrdU intensity vs. DNA content (PI intensity) for CTR or *Mgrn1*-null clones pulsed with BrdU (1 h), then chased for 2 or 4 h. For each plot, BrdU-positive cells are subdivided according to their DNA content in 2n (post-mitotic cells) and 4n (mitotic cells), using the gating scheme indicated by boxes in the figure. (**E**) Quantification of the percentage of post-mitotic and mitotic cells from the FACS analysis shown in (**D**). Color code as in panels **A** and **C**.

We assessed the expression levels of MGRN1 along the different phases of the cycle. Cells were synchronized in G0-G1 by serum deprivation, in early S phase with hydroxyurea (HU) or thymidine and in G2/M with colcemid. For HU, two conditions were employed, a 12 h treatment, and the same treatment followed by wash-off and further incubation in HU-free medium for 1 h. Since long treatments with HU cause irreversible replication fork stalling and fork collapse [41] these two conditions should yield similar results. Compared with asynchronous cells, MGRN1 levels were slightly lower in cultures enriched in G1 cells and augmented significantly upon transition from G1 to S phase (thymidine- or HU-treated cells). Cells arrested in G2/M with colcemid showed the same MGRN1 levels as control cells (Figure 3D). HU inhibits ribonucleotide reductase, leading to exhaustion of the deoxyribonucleotide pool and stalling of replication forks. Although long HU treatments cause irreversible fork collapse, shorter treatments are normally reversible, as DNA synthesis can resume and stalled forks restart when the drug is removed from the culture medium [41], resulting in increased fractions of cells in S phase during recovery. We followed the kinetics of cell cycle changes and MGRN1 accumulation in melan-a6 cells treated with HU for 4h, followed by wash-off and recovery in HU-free medium. Upon HU release, the percentage of cells in S phase increased during the first 4 h after wash-off. MGRN1 levels also rose over the first 3–4 h, then dropped towards control levels (Figure 3E). Interestingly, upon treatment with HU we observed a clear change of the staining pattern of HA-labeled MGRN1 isoform S (+) expressed in melan-a6 cells. MGRN1 was predominantly cytosolic in control cells, but HU treatment caused a significant translocation to nuclei, thus increasing the contribution of nuclear MGRN1 to the total intensity of immunostaining (Figure 3F). Therefore, MGRN1 expression and maybe its nuclear compartmentalization, apparently fluctuated in a cell cycle-dependent manner, being maximal during S phase. It should be noted that the presence of a N-terminal HA tag in the MGRN might interfere with myristoylation of glycine residue in position 2 of MGRN1. This post-translational modification, apparently targeting MGRN1 to mitochondria, has been recently described [42]. Therefore, it is possible that our HA-labeled MGRN1 variant might have an altered subcellular compartmentalization. However, this variant bears the nuclear localization signals encoded by exon 12 and our previous data showed that it is able to traffic between the cytosol and the nucleus [13]. Accordingly, it appears suitable to follow the nucleocytoplasmic trafficking of MGRN1, although our results should be interpreted with caution. We next analyzed the kinetics of progression through S phase of synchronized cultures. We compared the percentage of control and *Mgrn1*-KO cells in S phase at different times following release from cell cycle block at the G1/S interphase with HU (Figure 4A).

As expected, the fraction of cells in S phase immediately after HU washoff was higher for the three *Mgrn1*-KO clones, at all the time points analysed. Nevertheless, the kinetics of progression upon release of the block were similar for control and *Mgrn1*-KO cells. We probed S phase progression with higher sensitivity by pulse-chase experiments. Control and *Mgrn1*-KO cells were treated with HU as above, washed off, pulsed with 5-bromo-2′-deoxyuridine (BrdU) to label newly synthesised DNA, and analyzed for total (based on propidium iodide fluorescence) and newly synthesized DNA (based on BrdU staining) for up to 4 h in the presence of the microtubule depolymerizing agent colcemid to block mitosis (Figure 4B). The fraction of BrdU-labeled cells in early or mid S phase was then estimated (Figure 4C). We did not include in this analysis cells in late S phase, as the experimental setup did not allow for an adequate discrimination of these cells and cells in G2 and M phases. As shown in Figure 4B,C, the kinetics of DNA synthesis were comparable for control cells and *Mgrn1*-KO cells.

Since MGRN1 has been suggested to modulate microtubule stability and mitotic spindle orientation [24,25,26] we reasoned that its knockdown might lead to perturbation of the mitotic spindle checkpoint. To test this possibility, unperturbed cells were pulsed for 1 h with BrdU, then chased for up to 4 h and analyzed by flow cytometry for newly synthesized DNA (BrdU staining) and total DNA (propidium iodide staining) (Figure 4D). Mitotic and post-mitotic cells were identified as BrdU-positive cells having 4n and 2n DNA content, respectively (see scheme in Figure 4D). As shown in Figure 4D and 4E, control and *Mgrn1*-KO cells completed mitosis at a comparable rate, arguing against significant activation of the mitotic checkpoint in the absence of MGRN1. Similar results were obtained upon downregulation of MGRN1 expression in melan-a6 cells by siRNA treatment (Figure S3C). Overall, the data reported above showed that ablation of MGRN1 by CRISPR/Cas9 had a small but detectable effect on cell cycle progression, with accumulation of cells in S phase but little if any perturbation of the rate of DNA synthesis or the mitotic checkpoint.

### 2.3. Increased Genomic Instability in Cells Lacking MGRN1

Progress from G1 to S and throughout the DNA synthesis phase is heavily dependent on the occurrence of DNA damage, which activates a complex signaling network, the DNA damage response (DDR), to delay or stop cell cycle progression. Double strand breaks (DSBs) are highly cytotoxic lesions that efficiently trigger the DDR. DSBs can be caused by chemical or physical insults, such as ionizing radiation or oxidizing agents, and are also spontaneously formed in S phase by perturbations of the replication machinery [43].

**Figure 5:**
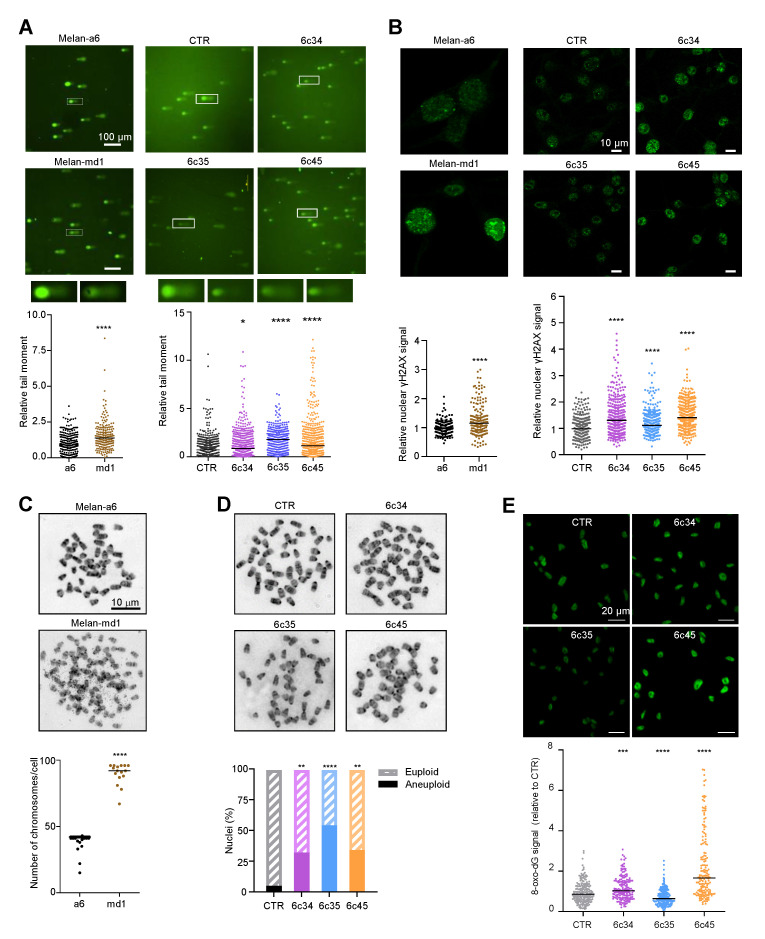
Genomic instability in *Mgrn1*-null cells. (**A**) Representative comet assays performed with melan-a6 and melan-md1 cells (left) or *Mgrn1*-KO and control cells (CTR, right). Quantitative analysis of at least 100 randomly selected comets was performed using CASPLAB software. Histograms show the mean average of the tail moment of *Mgrn1*-null cells relative to controls (*n* = 5 independent experiments, each one with at least 100 comets analyzed). Representative images of comet tails at 40× magnification are shown above histograms. (**B**) γ-H2AX immunostaining of melan-a6 control cells and melan-md1 cells (left), and *Mgrn1*-KO and control cells (right). Histograms show γ-H2AX nuclear intensity of *Mgrn1*-null cells relative to the corresponding control cells. (**C**) Metaphase chromosome spreads of melan-a6 and melan-md1 cells. The dot plot illustrates numerical chromosome aberrations in *Mgrn1*-null cells in > 10 independent spreads. (**D**) Same as in C for *Mgrn1*-KO clones. The graph represents the percentage of euploid (shaded) and aneupolid (solid) nuclei in *Mgrn1*-null cells compared to control cells. (**E**) Detection of 8-oxodG in parental melan-a6 cells (CTR) and *Mgrn1*-KO clones. Cells were stained with an anti-8-oxodG monoclonal antibody, observed in a confocal microscope and the intensity of the 8-oxodG stain was quantified with ImageJ. The upper images correspond to representative fields, and the lower graph represents the relative intensity of the 8-oxodG. The experiment was repeated twice, and at least 100 cells were analyzed in each case. * indicates *p* < 0.05, **, *p* < 0.01, ***, *p* < 0.001 and ****, *p* < 0.0001.

Since the DDR and the regulation of cell cycle progression are intimately linked and share a substantial part of their molecular machineries, it was of interest to analyze *Mgrn1*-KO cells for the presence of DNA breaks. We compared DNA integrity in control and *Mgrn1*-null cells using comet assays. Quantitative tail moment measurements revealed a higher abundance of DNA breaks in *Mgrn1*-null cells (Figure 5A). Upon formation of DSBs, histone H2AX is rapidly phosphorylated to yield γH2AX, a key early step in DSB repair. Accordingly, γH2AX is an indicator of DNA DSBs. In agreement with the comet assays, melan-md1 and *Mgrn1*-KO cells displayed stronger γH2AX labelling than control cells (Figure 5B). Moreover, karyotype analysis indicated that whereas few mitotic spreads from wildtype melan-a6 cells showed numerical abnormalities, ~80% of melan-md1 cells had chromosome numbers much higher than expected (Figure 5C). A statistically significant increase in the percentage of aneuploid cells was also found in *Mgrn1*-KO clones compared to control cells (Figure 5D). On the other hand, mitochondrial dysfunction in MGRN1-KO cells likely augments the production of reactive oxygen species (ROS), leading to higher oxidative stress [7]. In DNA, ROS predominantly damage bases or deoxirribose, with 8-oxo-7,8-dihydroguanine (8-oxodG) as the main oxidation product [44]. We compared the levels of 8-oxodG in melan-a6 and *Mgrn1*-KO clones. These were quite variable, and even lower in one of the clones relative to controls (Figure 5E), since they varied in the order 6c35 < control melan-a6~6c34 < 6c45. Accordingly, base oxidation in nuclear DNA of *Mgrn1*-KO clones was not as marked as accumulation of DNA strand breaks, that was observed in all cases. Therefore, the high burden of strand breaks in *Mgrn1-*KO cells could not be accounted for merely on the basis of faster formation due to higher oxidative damage.

**Figure 6:**
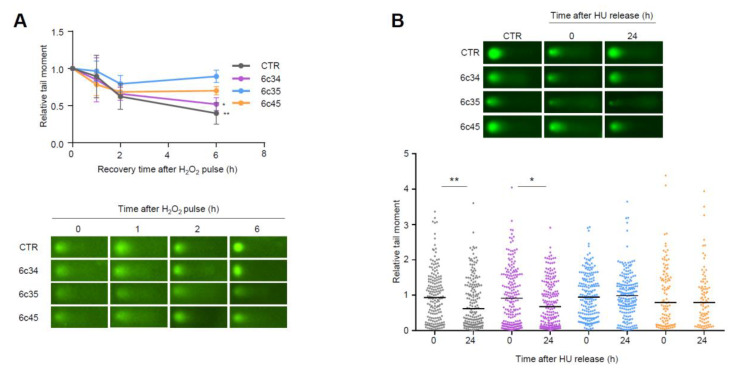
Impaired repair of DNA strand breaks in *Mgrn1*-KO melanocytes. (**A**) Clearance of DNA breaks generated by an oxidative challenge. *Mgrn1*-KO cells or the corresponding control were challenged with Luperox (150 μM, 10 min, 4 °C), washed and allowed to recover for the times shown in peroxide-free medium. The presence of DNA breaks was estimated by means of comet assays. The graph indicates the evolution of tail moments, normalized to the value at time 0 (*n* = 3). A representative comet for each experimental condition is shown below. (**B**) Clearance of HU-induced lesions in *Mgrn1*-KO cells. Cells were treated with HU (2 mM, 6 h), then allowed to recover in HU-free medium for 24 h. The presence of DNA breaks after the treatment with HU (time 0) or after a 24 h recovery was analyzed by comet assays, as in (**A**). * indicates *p* < 0.05, **, *p* < 0.01.

Higher steady-state levels of DNA lesions can result from an increased rate of formation and/or a decreased rate of clearance by DNA repair processes. We analyzed the kinetics of repair of DNA strand breaks generated by exogenous agents. To this end, we challenged control or *Mgrn1*-KO cells with a short and intense oxidative pulse (Luperox 150 μM, 10 min, 4 °C) previously shown to result in formation of strand breaks in melanocytes [45], then allowed them to recover for up to 6 h in peroxide-free medium at 37 °C. Clearance of DNA breaks was followed by comet assays. As shown in Figure 6A, the rate of break clearance was lower for *Mgrn1*-KO cells.

To extend these data, we analyzed the repair of breaks generated upon prolonged replicative stress caused by long HU treatment (6 h). Within a 24 h recovery, control melan-a6 cells were able to repair most of the ensuing DNA breaks. Conversely, one clone of *Mgrn1*-KO cells exhibited a lower but still significant repair capacity, and the other two clones under study did not achieve a statistically significant clearance of the HU-induced lesions (Figure 6B). Of note, these clones (6c35 and 6c45) were also less efficient in repairing DNA strand breaks generated by an oxidative pulse, and exhibited the highest levels of DNA damage under basal conditions. Therefore, lack of MGRN1 expression decreased the ability of melanocytes to cope with strand breaks generated by oxidizing agents or by HU-induced replicative stress. This might explain, at least partially, the genomic instability in *Mgrn1*-KO cells.

### 2.4. Effects of MGRN1 Ablation on Growth and Invasive Properties of Melanoma Cells

**Figure 7:**
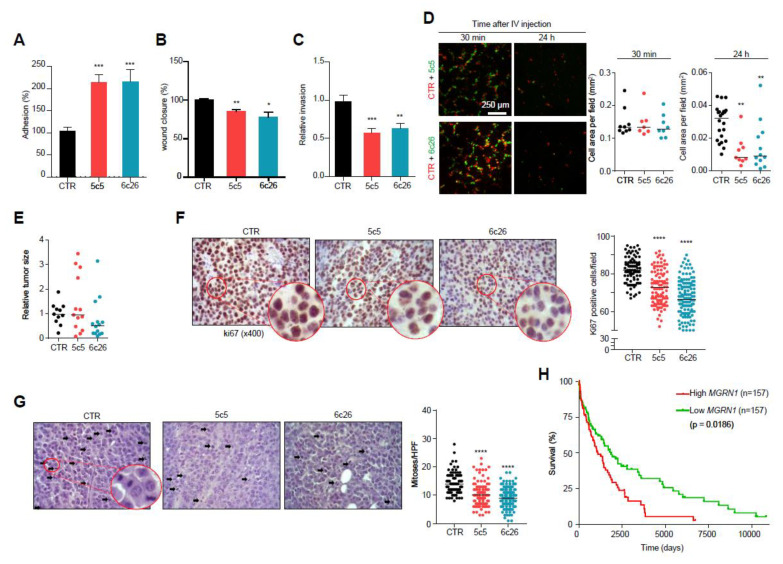
Modulation of melanoma cells adhesion, migration and invasive properties by MGRN1. (**A**) Percentage of adhesion to a collagen I matrix of *Mgrn1*-KO B16F10 clones (5c5, 6c26) relative to control cells. (**B**) Wound closure of *Mgrn1*-KO B16F10 cells after scratch assay (24 h). Results expressed as the percentage of wound closure relative to control cells. (**C**) Decreased invasion through collagen I after 24 h of *Mgrn1*-KO B16F10 clones relative to melan-a6 control cells. (**D**) Short term lung colonization assay. Micrographs on the left correspond to representative confocal microscopy images of the lungs of mice sacrificed 30 min or 24 h after injection in the tail vein of equal numbers of control (red) and *Mgrn1*-KO (green) B16F10 cells. Dot plots on the right show the cell area occupied by control (CTR) or the indicated *Mgrn1*-KO cells, as a measure of the relative abundance of each cell type. (**E**) Relative size of tumors derived from control or *Mgrn1*-KO B16F10 cells intradermally injected into the flanks of C57BL/6J mice, and analyzed 18 days after injection. At least 4 mice per group were analyzed, and the experiment was repeated 3 times. (**F**) Immunohistochemical staining of ki67 in tumors grown as in (**E**). Representative micrographs (left) show zoomed areas for better comparison of the relative intensities of the stain. The dot plot on the right shows the percentage of ki67-positive cells in control and *Mgrn1*-KO cells. (**G**) Estimation of mitotic index in tumors grown as above. Representative migrographs of haematoxylin/eosin-stained preparations were counted for mitotic figures (highlighted with arrows). The mitotic index was estimated by the median of the number of mitoses in 10 high power fields (HPF, X400), and is shown in the dot plot on the right. (**H**) Kaplan-Meier curves for survival of melanoma patients, according to data from the TCGA database. Patients were stratified as a function of high and low MGRN1 levels in primary tumor specimens (*n* = 157 in each case). * indicates *p* < 0.05, **, *p* < 0.01, ***, *p* < 0.001 and ****, *p* < 0.0001.

So far, we have shown that knockdown of MGRN1 in normal melan-a6 melanocytes (i) induced a more differentiated cellular morphology, (ii) increased cell adhesion to collagen matrices and impaired 2D and 3D motility, (iii) altered cell cycle progression and (iv) led to genomic instability with accumulation of DNA damage. Similar changes might have important implications for the behavior and aggressiveness of melanoma cells. To test this possibility, we knocked down MGRN1 expression in B16-F10 melanoma cells by CRISPR/Cas9 and selected two independent *Mgrn1*-KO clones for further analysis (Figure S4A–C). These clones phenocopied the effects of MGRN1 ablation in melan-a6 melanocytes. As previously shown for *Mgrn1*-KO melan a6-cells, the proliferation rate of B16-derived *Mgrn1*-KO clones was not significantly different compared with control cells (Figure S4D). Indeed, *Mgrn1*-KO B16 melanoma cells were more differentiated as shown by higher melanin contents (Figure S5A), and presented higher levels of DNA damage as shown by comet assays, γH2AX staining and altered ploidy (Figure S5B–D). Moreover, B16 *Mgrn1*-KO clones displayed significantly increased cellular adhesion to collagen I (Figure 7A), and impaired migration in 2D scratch assays (Figure 7B) and 3D collagen I invasion assays (Figure 7C).

We also performed short-term lung colonization assays. *Mgrn1*-KO and control B16F10 cells were labelled with green or orange fluorescent dyes respectively, and co-injected in equal numbers in the tail vein of C57BL/6J mice. Mice were sacrificed after 30 min or 24 h and the lungs were examined for fluorescent cells. Thirty min after inoculation, comparable numbers of control and *Mgrn1*-KO cells were present in the lungs. However, significantly fewer *Mgrn1*-KO cells remained 24 h after the injection, indicative of impaired colonization potential upon repression of MGRN1 (Figure 7D). On the other hand, tumors generated by intradermal injection of *Mgrn1*-KO cells showed a numerically lower mean size than those derived from control B16 cells, but the differences did not reach statistical significance due to the high dispersion of the data (Figure 7E). Nevertheless, a lower rate of tumor growth was also suggested by immunochemical staining of the proliferation marker Ki67, which was stronger in tumors arising from control cells (Figure 7F). Moreover, estimation of the mitotic index in haematoxylin/eosin-stained preparations revealed a significantly lower number of mitotic cells in *Mgrn1*-KO tumors (Figure 7G). These in vivo experiments showed that, at least in the B16 mouse melanoma model, MGRN1 expression may regulate the malignant phenotype, with effects on proliferation, adhesion, motility, lung colonization potential and maybe also on the rate of tumor growth. Accordingly, it was of interest to analyze a possible relationship of MGRN1 expression and survival of melanoma patients. For this purpose, we interrogated the TCGA database for correlations of high and low MGRN1 mRNA levels and clinical data, in a set of >450 melanoma patients (Figure 7H). High and low expression tumors were defined as the upper and lower 33% MGRN1-expressing tumors, respectively. Kaplan-Meier curves showed that low expression of MGRN1 in tumors was significantly associated with better survival. Therefore, MGRN1 expression might be an important determinant of the phenotype and aggressiveness of human melanoma cells.

## 3. Discussion

The multiple pathological implications of the *mahoganoid* mouse mutation clearly demonstrate that MGRN1 plays important physiological functions [5,46]. These pathological effects of MGRN1 loss have been related with mitochondrial dysfunction with increased oxidative stress [7], aberrant microtubule dynamics and stability with altered mitotic spindle orientation [24,25,26], defects of microtubule-dependent transport of intracellular organelles [21], deregulation of the transcriptional response to proteotoxic stress [30], and others. However, in spite of its relationship with components of the cytoskeleton, no study on the regulation by MGRN1 of cell shape and movement of normal or cancer cells has yet been reported. Moreover, the likely roles of MGRN1 in maintaining genomic stability and normal cell cycle progression have not been explored, in spite of the potential genotoxic effects of oxidative stress and/or mitotic spindle missorientation. Here we showed that *Mgrn1* knockdown in normal melanocytes and melanoma cells induced a phenotype characterized by: (i) cell morphology changes with increased size, higher number of dendrites, longer protrusions and higher melanin content, (ii) impaired cell motility and higher adhesion to collagen matrices, (iii) abnormal cell cycle progression with increased percentage of cells in S phase, and (iv) decreased genome stability with accumulation of DNA damage.

Concerning genomic stability, ablation of MGRN1 led to higher abundance of DSBs as demonstrated by staining of γH2AX foci and comet assays. Melanocytic cells lacking MGRN1 are likely exposed to a higher oxidative stress due to mitochondrial dysfunction [7,42] as well as increased melanogenesis [47], since ROS are generated at several steps of the melanogenic pathway [48,49]. Conceivably, an augmented formation of DNA breaks resulting from enhanced ROS production might thus contribute to the higher steady-state level of these lesions in *Mgrn1*-null melanocytes and melanoma cells. However, the burden of DNA breaks in MGRN1-KO cells did not correlate with the abundance of 8-oxodG, the prevailing product of DNA base oxidation and therefore a good marker of DNA exposure to ROS. Moreover, strand breaks caused by an exogenous oxidative challenge or following replication fork stalling by HU were less efficiently cleared in the absence of MGRN1, thus suggesting that defective break repair also contributed to the high burden of DNA breaks in *Mgrn1*-KO cells. Of note, comparison of the rates of break clearance in three individual *Mgrn1*-KO clones showed that the lower the residual repair capacity of the clone, the higher the basal levels of DSBs in unperturbed cells. These data support the notion that MGRN1 might be involved in the detection and/or repair of breaks in DNA, and that accumulation of DNA damage in *Mgrn1*-null cells might be accounted for mainly by inefficient clearance of these lesions. Within this context, it will be interesting to find out whether all the MGRN1 isoforms contribute to the protection against DNA damage or, alternatively, if this action is specific to particular isoforms such as those bearing a nuclear localization signal. It will also be important to learn if the E3 ligase activity of MGRN1 is required. Of note, at least two proteins reported to modulate DNA damage, ARRB1 [50] and TSG101 [51] are likely ubiquitinated by MGRN1 [33,51]. Further work is required to establish if these or other MGRN1 substrates contribute to increased DSB repair potential in MGRN1-expressing cells. In any case, it can be speculated that in cells lacking MGRN1, this impairment of DSB repair would lead to progressive accumulation of DNA damage, since breaks occurring normally during the S phase of the cell cycle would be less efficiently repaired. This might account, at least partially, for the milder phenotype of *Mgrn1*-KO cells newly derived from melan-a6 or B16 cells, as compared with melan-md1 cells derived from *mahoganoid* mice. Interestingly, human MC1R, a GPCR that activates DNA damage repair in melanocytes [52,53,54,55] (reviewed in [56,57]) interacts with MGRN1 and promotes its nuclear localization [13]. Given that activation of MC1R in human melanoma cells stimulates the clearance of DNA strand breaks generated by oxidative stress [45], a possible involvement of MGRN1 in this MC1R-dependent genoprotective action cannot be ruled out and deserves further analysis.

In summary, a lower DNA repair efficiency and possibly a faster rate of DSB formation may contribute to increased levels of DNA fragmentation in *Mgrn1*-null cells. The resulting activation of a DDR might interfere with cell cycle progression, thus accounting for the higher fraction of cells in S phase. The slightly disturbed cell cycle kinetics in *Mgrn1*-KO cells is consistent with a genome-wide functional screening for human cell cycle regulators, which found increased numbers of cells in S phase in MGRN1-silenced U2OS cells [40]. Moreover, a recent RNA-sequencing analysis of genes regulated by MGRN1 after proteasomal stress identified “cell cycle phase” as the biological process with the most significant gene ontology functional enrichment score [30]. In any case, the effect of MGRN1 ablation on progression through S phase was subtle according to our results. Pulse-chase experiments where newly synthesized DNA was labeled with BrdU did not reveal substantial alterations of the rate of DNA synthesis or completion of mitosis. Of note, the impact of DDR on cell cycle progression is heavily dependent on activation of TP53 [58]. The studies reported here were performed in cells with alterations in this pathway since B16 melanoma cells lack expression of functional TP53 [59] and melan-a6 and melan-md1 cells were derived from mice heterozygous for a *Cdkn2a* deletion [36]. Therefore, it can be speculated that the DDR induced by accumulation of DNA damage in MGRN1-deficient cells might have a higher impact on cell cycle progression in cells bearing an intact ARF/TP53 module, although this hypothesis remains to be proven.

Conceivably, genomic instability, which is a major driver of tumorigenesis, might promote the malignant transformation of *Mgrn1*-null cells. However, a higher rate of melanoma or other malignancies has not yet been reported for *mahoganoid* mice, and, with a rate of ~1.7% non-synonymous substitutions, *MGRN1* mutations are not frequent in human melanoma according to the TCGA database. This low mutation rate is nevertheless comparable to that of *MITF* (~2.3%), a gene known to be mutated in a subset of melanomas [60]. Moreover, a higher prevalence of *MGRN1* mutations is found in other tumor types such as endometroid cancers, with ~6% of these tumors in the TCGA cohort harboring non-synonymous *MGRN1* mutations. Therefore, the possibility that *MGRN1* might behave as a cancer susceptibility gene deserves further analysis. In this context, it will be interesting to analyze the rate of *MGRN1* mutations in melanoma families without mutations in known susceptibility genes such as *CDKN2A*, *CDK4*, *BAP1*, *TERT*, *POT1*, *ACD*, *TERF2IP*, and *MITF*. Interestingly, mutations in these genes are only found in 30–50% of melanoma families [61].

Whatever the contribution of MGRN1 to melanomagenesis, a role of its expression level in melanoma progression is likely, according to our data. In keeping with this possibility, a study on osteosarcoma patients showed recurrent amplifications of the 16p13 region where *MGRN1* is located [62], and another investigation reported hypomethylation of CpG sites in *MGRN1* in blood cells from breast cancer patients [63]. Here we showed that loss of MGRN1 expression in mouse melanocytes promoted a differentiated phenotype with increased pigmentation, dendricity and adhesion to collagen I on one hand, and decreased motility on the other. This phenotypic switch was independent on changes in MITF expression. Cellular adhesion is intimately connected with migration and invasion, and it is known that microtubules are key regulators of these processes [64]. Given that MGRN1 regulates microtubule dynamics through ubiquitylation of α-TUB [21] our findings of altered adhesion, migration and invasion in *Mgrn1*-null cells are not surprising. In agreement with these data, we found low rates of lung colonization following injection of *Mgrn1*-KO B16 melanoma cells in the tail vein of mice, compared with MGRN1-expressing cells. Importantly, complete knockdown of MGRN1 was not required to reduce the invasion potential of melanocytic cells, as a siRNA-mediated decrease in MGRN1 abundance to ~25% residual levels lowered invasion indexes on collagen I to >50% of control values. Concerning human melanoma, analysis of data in the TCGA database is consistent with our in vivo data in the B16 mouse model, as highlighted by the inverse relationship between *MGRN1* expression and patient survival. Moreover, a statistically significant correlation of low MGRN1 expression and higher survival probability was also observed for two prevalent types of cancer, namely lung squamous cell carcinoma (*p* = 0.0049) and ovarian cancer (*p* = 0.0029) according to the TCGA database. Nevertheless, no correlation was observed for the majority of datasets suggesting that the effect of MGRN1 levels on patient survival might be specific to a restricted set of tumor types. This indicates that the physiological relevance of MGRN1 might be critically dependent on the cellular context in spite of a relatively widespread expression, as previously suggested by others [32]. In any case our data strongly suggest that MGRN1 might be an important determinant of melanoma aggressiveness and warrant further studies to clarify its role in human melanoma.

## 4. Materials and Methods 

### 4.1. Reagents

Laboratory reagents and protease or phosphatase inhibitors were from Sigma (St. Louis, MO, USA), Calbiochem (Darmstadt, Germany), Merck (Darmstadt, Germany) or Prolabo (Barcelona, Spain), unless specified otherwise. Lipofectamine 2000 and Opti-MEM I were from Invitrogen (Carlsbad, CA, USA). Reagents for SDS-PAGE and Western blot were from Bio-Rad (Richmond, CA, USA). 

### 4.2. Cell Culture

Cell culture reagents were from Gibco (Gaithersburg, MD, USA). Mouse melan-md1 and melan-a6 cells were obtained from the Wellcome Trust Functional Genomics Cell Bank and were grown in RPMI 1640 medium enriched with 10% fetal bovine serum (FBS), 200 nM 12-O-tetradecanoylphorbol-13-acetate (TPA), 100 U/mL penicillin and 100 μg/mL streptomycin sulphate. Mouse melanoma B16F10-luc cells were a gift from Prof. JN Rodríguez-López (University of Murcia, Spain) and were grown in DMEM enriched with 10% FBS, 100 U/mL penicillin and 100 μg/mL streptomycin sulphate. Cells were cultured for a maximum of five passages and their phenotype was verified in every experiment. For cell culture on thick layers of collagen I, collagen I matrices were prepared using atelopeptide fibrillar bovine dermal collagen (PureCol, Advanced BioMatrix, San Diego, CA, USA) at a final concentration of 1.7 mg/mL in DMEM and allowed to polymerize for 4 h. Cells were imaged after 24–48 h in culture in 1% serum media.

### 4.3. Expression Constructs and Transfection

HA epitope-tagged human MGRN1, isoform S(+), UniProt number O60291-1, was cloned between the *Hind*III and *Eco*RI sites of the pcDNA3.1 expression plasmid. To this end, the wildtype cDNA sequence cloned in the same vector as previously published [13] was amplified with primers CCCAAGCTTATGTACCCATACGATGTTCCAGATTACGCTGGCTCCATT (forward) and CGTGAATTCTTACTCGTCTATACCAACAGAGC (reverse), where the sequence corresponding to the restriction sites is underlined. The cloned open reading frame was verified by automated sequencing of both strands. Cells were transfected with Lipofectamine 2000 according to manufacturer’s instructions.

### 4.4. RNAi Transfection 

Mouse melanocytic cells (2 × 10^5^) were seeded in a 6-well plate and transfected the next day with individual mouse *Mgrn1* siRNA oligonucleotides siGENOME 1 or 4, or a stoichiometric mixture of these oligonucleotides, at a final concentration of 30 nM, using Opti-MEM (Invitrogen) and 5 µL/well DharmaFECT 4 Transfection Reagent from Dharmacon (Lafayette, CO, USA). Non-targeting siRNA #2 was used as control. Forty-eight h later, cells were used for the desired experiment. siRNA sequences are specified on Table S1.

### 4.5. Generation of CRISPR/Cas9-Based Mgrn1-KO Cells

Target sequences for CRISPR-RNA from Dharmacon are specified on Table S2. Efficiencies and potential off-targets were determined using the *Breaking-Cas* web server (http://bioinfogp.cnb.csic.es/tools/breakingcas) [65]. Cells were co-transfected with 2.5 µg Cas9 Nuclease Expression plasmid with puromycin selection marker, 50 nM trans-activating CRISPR RNA (tracrRNA), 50 nM crispr-RNA5 or crispr-RNA6 and 6 µg/mL DharmaFECT Duo Transfection Reagent (all from Dharmacon). For negative control cells, we transfected 50 nM crRNA Non-targeting Control #1. After incubation at 37 °C for 72 h, puromycin-resistant clonal cells were selected. Confirmation and selection of positive clones was performed by automated sequencing after PCR amplification of a 700-pb fragment on exon 1 using genomic DNA as template.

### 4.6. Melanin Content Measurement

Melanin content was determined after alkaline solubilisation using a colorimetric assay [66]. Data were presented as relative melanin content per microgram of protein.

### 4.7. 3D Spheroid Assays

Spheroid cell culture was performed using the hanging drop method, adapted from Del Duca et al. [67]. Briefly, spheroids of melan-a6 or melan-md1 cells were collected and resuspended in a collagen I solution (1.7 mg/mL in DMEM), added on top of a thin layer of previously polymerized collagen I. For growth quantification, increase of the area of spheroids between day 0 and 4 was calculated using ImageJ.

### 4.8. In Vitro Cellular Adhesion 

Adherent cells were detached using a non-enzymatic cell dissociation solution and seeded on a thick layer of Collagen I (1.7 mg/mL) prepared as mentioned above or on a thin layer of Matrigel (20 µL at 7–9 mg/mL; ECM Gel, from Sigma) polymerized on top of a thick layer of collagen I (60 µL at 1.7 mg/mL) in a 96-well plate. Percentage of adhering cells was calculated as the number of cells remaining after washing.

### 4.9. 2D and 3D Migration Assays 

For 2D migration assays, an artificial gap was made by scratching a confluent cell monolayer and cells were incubated in 1% FBS-containing media. The closure of this gap was monitored at time 0 and after 24 h. The area of the gap at both time points was measured using ImageJ and data were presented as % wound closure. In 3D migration assays, cells were embedded in serum-free bovine collagen I (2.3 mg/mL) to a final concentration of 1.5 × 10^4^ cells/100 μL in a 96-well plate and processed as previously described [68]. Quadruplicates per condition were performed. A 3D migration index was calculated as number of migrating cells at 50 μm/total number of cells.

### 4.10. Cell Proliferation Assays

Three independent methods were used. For manual counting, cells were seeded and manually counted using a hemocytometer at the different time points from 24 to 96 h. Normalized cell number values were plotted using GraphPad Prism (GraphPad Software, San Diego, CA, USA, www.graphpad.com) and doubling times were calculated by nonlinear regression using an exponential growth equation. A cell labelling assay was performed by labelling cells with CellTrace^™^ (CFSE, Invitrogen) followed by flow cytometric measurements of the fluorescence intensity of cells using a FACScan system (Becton Dickinson, Franklin Lakes, NJ. USA), 488 nm excitation and 517 nm emission). Arbitrary units of fluorescence normalized with fluorescence intensity at time 0 h were plotted using GraphPad Prism and half-life values were obtained by nonlinear regression using a one phase decay equation. Finally, an MTT assay was also employed. Cells were incubated with MTT solution and absorbances at 570 nm and at 690 nm (background) were measured using an ELx800™ microtiter plate reader (BioTek™, Winooski, VT, USA). Normalized absorbance values were plotted using GraphPad Prism and doubling times were calculated as for the manual counting assay.

### 4.11. Cell Cycle Analysis

Cells were fixed in ethanol 70% in PBS, then pelleted and resuspended in PBS containing 100 µg/mL RNase A and 40 µg/mL propidium iodide (PI). Cells were analysed in a FACScanto cytometer from BD Biosciences (San Jose, CA, USA). The kinetics of cell cycle progression were analysed according to [69] by pulse-chase experiments using cells labelled with BrdU (20 µM, 1 h), then chased for different times in the presence or absence of colcemid (0.1 µg/mL). Cells were labelled with PI and analysed as above. For experiments of HU-mediated interference, cells were incubated in complete medium containing HU at a final concentration of 2 mM for the required times, washed with PBS and further incubated in HU-free medium for various times, as indicated in the corresponding Figures. For pulse-chase experiments, cells were treated with 2 mM HU for 3 h, washed with PBS and allowed to recover for 1 h in fresh medium. Then, BrdU and colcemid were added (final concentrations 20 µM and 0.1 µg/mL respectively). Cells were collected at the required times and resuspended in cold PBS. 1 × 10^6^ cells were resuspended in 100 µL of ice-cold PBS/1% FBS, then added dropwise to 5 mL of −20 °C, 70% ethanol and kept overnight at 4 °C. The fixative was removed, 1 mL of 2 N HCl/Triton X-100 was added dropwise and cells were centrifugated, neutralized with 0.1M sodium tetraborate, pH 8.5 and centrifugated again. The pellet was resuspended with 75 µL of BrdU staining mix (50 µL 0.5% Tween 20 in PBS containing 1% BSA, 20 µL FITC conjugated anti-BrdU, 50 µg RNAse) and incubated at room temperature for 45 min. Samples were pelleted, resuspended in 0.5 mL of PBS containing 10 µg/mL propidium iodide and were analyzed using a FACScanto cytometer as described above.

### 4.12. Immunoblotting

Western blotting was performed as described [13,33] using antibodies specified in Table S3. Quantification of band intensities was performed with ImageJ and data are always given after normalization to a loading control.

### 4.13. Confocal Fluorescence Microscopy Imaging 

Cells were fixed in 4% formaldehyde, permeabilized and blocked in BSA. F-actin was stained using Phalloidin eFluor-570 (eBioscience, Paisley, UK) and DNA was stained with DAPI. Images were taken with a SP8 Leica laser scanning confocal microscope and software (Leica Microsystems GmbH, Wetzlar, Germany). Backscattered light (reflectance) was collected to image the matrix surrounding cells. For cMyc and γ-H2AX staining, cells were processed as described in [45]. Images were taken with a SP8 Leica laser scanning confocal microscope and software (Leica Microsystems GmbH) with HCXPL APO CS 40x or 63x objective lenses. Nuclear cMyc and γ-H2AX fluorescence signal was quantified by calculating the pixel intensity in single cell nuclei relative to the nucleus area. At least 200 randomly selected cells per condition were quantified using ImageJ.

### 4.14. Comet Assays 

The Alkaline Comet Assay was performed according to the manufacturer’s protocol (Trevigen, Gaithersburg, MD, USA) and as described in [45].

### 4.15. Chromosomal Spreads

Cells were incubated with Colcemid Solution (200 ng/µL, Gibco, Madrid, Spain), treated with a hypotonic solution (2.5 mL, 0.055 M KCl) at 37°C for 30 min and fixed with fresh Carnoy’s Fixative (3:1 ratio of methanol/glacial acetic acid). Cell suspensions were dropped on slides and dried at 60 °C, 12 h. Slides were stained with freshly prepared Giemsa Solution (7% Giemsa Stain, Sigma GS-500, in KH_2_PO_4_ Buffer, pH 6.8) after a 0.15% trypsin treatment. An average of 10 images per condition were taken.

### 4.16. Construction of Kaplan-Meier Curves for Patient Survival as a Function of MGRN1 Expression

We used the GDC TCGA Melanoma (SKCM) cohort of the TCGA database (https://portal.gdc.cancer.gov/) for analysis of the correlation of high and low MGRN1 mRNA levels with patient survival. This dataset comprises gene expression and clinical data for >450 melanoma patients. High and low expression tumors were defined as the upper and lower 33% MGRN1-expressing tumors. Kaplan-Meier survival curves were obtained with GraphPad Prism and were compared using a log-rank test.

### 4.17. In Vivo Experiments

Experiments with mice met the Animal Welfare guidelines and followed protocols approved by the Ethics Committee of the University of Murcia (approval code: 482/2018). For tumor growth assays, 2.5 × 10^5^ *Mgrn1*-KO B16F10-luc melanoma cells were intradermally injected into the flanks of C57BL/6J mice. Mice were sacrificed and scanned using a computed tomography (CT) equipment (Albira, Bruker, Billerica, MA, USA). Tumor volume was calculated from the images taken with the scan. Tumors and peripheral organs (lungs and liver) were excised, fixed and paraffin-treated for immunohistochemical analysis. This analysis included estimation of the number of mitotic cells/field using hematoxylin/eosin-stained preparations and evaluation of the percentage of cells positively stained for mKi67. 

For short-term lung colonization assays, *Mgrn1*-KO and control B16F10-luc cells were fluorescently labelled with either cell-permeable CellTracker Green CMFDA Dye (10 µM) or Orange CMTMR Dye (15 µM). Equal numbers of MGRN1-depleted and control cells (5 × 10^5^ cells in 0.3 mL PBS, ratio 1:1) were co-injected in the tail vein of C57BL/6J mice. Mice were sacrificed after 30 min or 24 h. Lungs were examined for fluorescent cells under a confocal microscope (Leica TCS) equipped with a PL APO 10X/0.40 AN objective lense and Leica Software. An average of 15 images per mouse were taken. Data were presented as cell area/field/mouse. Cell area per field was calculated as the average of cell area covered by fluorescence per image using ImageJ.

### 4.18. Histological Analysis

Samples were fixed in 4% neutral buffered formalin (Panreac Quimica), then paraffin embedded sections were stained with standard hematoxylin/eosin stain. To determine the proliferative index of tumors, an indirect ABC immunohistochemical procedure was employed and samples were stained for Ki67. Mitotic index was estimated by the median (±standard deviation) of the number of mitoses in 10 high power fields (HPF, X400), according with recommendations of the American Joint Committee on Cancer staging system. The proliferative index was estimated by the count of positive cells in 10 HPF (100 tumoral cells per field) and expressed by the percentage of the median (± SD). Examinations were performed using a direct light microscope (Zeiss Axio Scope A10, Karl Zeiss, Madrid, Spain) with a digital camera (Axio icC 3, Karl Zeiss) and using a digital image analysis software (Axio Vision SE64, Ver. 4.9.1).

### 4.19. Statistical Analysis

All analyses were carried out using GraphPad Prism. For comparison of melan-md1 and melan-a6 cells, unpaired two-tailed Student’s *t*-tests were performed and data are given as mean ± standard error of the mean (sem) unless indicated otherwise. For comparison of CRISPR/Cas9 clones and control cells transfected with a non-targeting crRNA, one-way ANOVA with Tukey post-test (for multiple comparisons) and the Chi-square test for contingency graphs were performed, and data are given as mean ± sem. Scatter dot plots were analyzed with the Mann-Whitney test for comparison of 2 groups or the Kruskal-Wallis test with Dunn’s post-test for multiple comparisons, and the median is indicated. *p* values of less than 0.05 were considered statistically significant. * indicates *p* < 0.05, **, *p* < 0.01, ***, *p* < 0.001 and ****, *p* < 0.0001.

## 5. Conclusions

Knockdown of the E3 ubiquitin ligase MGRN1 in melanocytes decreased their 2D and 3D motility, and increased their adhesion to collagen and the fraction of cells in the S phase of the cell cycle. Expression of MGRN1 was required to maintain genomic stability, probably by allowing for efficient repair of DNA strand breaks. Knockout of MGRN1 in mouse melanoma cells caused similar changes and impaired their lung colonization potential. Consistent with the phenotypic changes in MGRN1-depleted mouse melanocytes and melanoma cells, analysis of the melanoma cohort of the TCGA database showed that low MGRN1 expression is significantly associated with better human melanoma patient survival. Accordingly, MGRN1 is an important determinant of the phenotype and aggressiveness of melanoma cells.

## Supplementary Materials

The following are available online at https://www.mdpi.com/2072-6694/12/10/2840/s1, Figure S1: Generation of clones of MGRN1-KO mouse melanocytes: strategy and validation, Figure S2: Changes in shape and motility of melan-a6 mouse melanocytes upon downregulation of MGRN1 expression with siRNA, Figure S3: Proliferation rates and cell cycle progression of MGRN1-depleted cells, Figure S4: Generation of MGRN1-KO B16 mouse melanoma cells: strategy, validation and effect on the rate of proliferation, Figure S5: Increased genomic instability in MGRN1-KO B16 mouse melanoma cells, Table S1. Sequence of control and *Mgrn1*-directed siRNA, Table S2. Details of crispr-RNAs for knockout of *Mgrn1* expression in mouse melanocytic cells, Table S3. Antibodies used. Uncropped original Western-blots.
