# Supplementary material for: Mahogunin Ring Finger 1 Is Required for Genomic Stability and Modulates the Malignant Phenotype of Melanoma Cells
Source: Cancers (Basel). 2020 Oct 1;12(10):2840. doi: 10.3390/cancers12102840 (PMC7599452; doi:10.3390/cancers12102840)

Supplementary Materials for

Mahogunin Ring Finger 1 is required for genomic stability and modulates the malignant phenotype of melanoma cells

Idoya Martínez-Vicente, Marta Abrisqueta, Cecilia Herraiz, Julia Sirés Campos, María Castejón Griñán, Dorothy Bennett, Conchi Olivares, Jose Carlos García-Borrón, Celia Jiménez-Cervantes

This file includes:

Supplementary figures S1 to S5 with captions
Supplementary Tables 1 to 3
All uncropped Western-blots

A

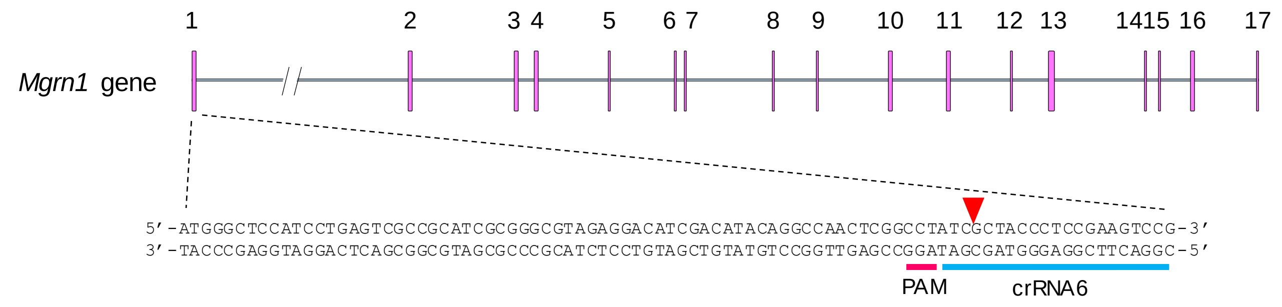

B

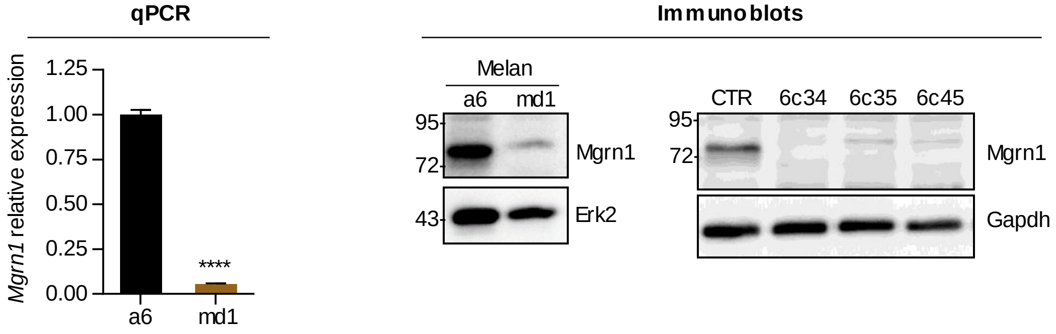

C

crRNA6 clones	Edited sequences on *Mgrn1* exon 1	
CTR	Alleles 1 and 2:	58AAC TCG GCC TAT **CG**C TAC CCT CCG AAG TCC GGA90
6c34	Alleles 1 and 2:	58AAC TCG GCC TAT **(CG)** C TAC CCT CCG AAG TCC GGA90
6c35	Allele 1:<br>Allele 2:	58AAC TCG GCC TAT **C (GC)** TAC CCT CCG AAG TCC GGA90<br>58AAC TCG GCC TAT **CGG**C TAC CCT CCG AAG TCC GGA90
6c45	Allele 1:<br>Allele 2:	58AAC TCG GCC TAT **C (G)** C TAC CCT CCG AAG TCC GGA90<br>58AAC (TCG GCC TAT **CGC** TAC CCT CCG A) AG TCC GGA90

```
              1       10        20        30        40        50        60        70        80      532
              |--------+---------+---------+---------+---------+---------+---------+---------|//------|
         CTR  MGSILSRRIAGVEDIDIQANSAYRYPPKSGNYFASHFFMGGEKFDTPHPEGYLFGENMDLNFLGSRPVQFPYVTPAPHEP//------|
        6c34  MGSILSRRIAGVEDIDIQANSAY-LPSEVRKLLCFAFFHGRREI
   6c35-iso1  MGSILSRRIAGVEDIDIQANSAY-LPSEVRKLLCFAFFHGRREI
   6c35-iso2  MGSILSRRIAGVEDIDIQANSAYRLPSEVRKLLCFAFFHGRREI
   6c45-iso1  MGSILSRRIAGVEDIDIQANSAYPTLRSPETTLLRIFSWEERNLTLPILKVTSLERTWT
   6c45-iso2  MGSILSRRIAGVEDIDIQANS-------PETTLLRIFSWEERNLTLPILKVTSLERTWT
```

Figure S1. Generation of clones of MGRN1-KO mouse melanocytes: strategy and validation.
(A) Schematic representation of *Mgrn1* highlighting the exon 1 sequence targeted by crRNA6. This 20 nt sequence pairs with the DNA target (blue bar on bottom strand), directly upstream of a 5’-NGG adjacent motif (PAM; in pink).
(B) Relative mRNA levels of Mgrn1 in melan-a6 and melan-md1 cells estimated by qPCR (left) and representative immunoblots for Mgrn1 in melan-a6, melan-md1 and Mgrn1-KO melan-a6 cells (right). Erk2 or Gapdh were used as loading control. Graph shows mean ± SEM and t-test was used for statistical analysis (****p<0.0001).
(C) Top: edited sequence (from nt 58 to 90) in exon 1 of *Mgrn1* in Mgrn1-KO clones 6c34, 6c35 and 6c45, compared with the consensus sequence in control (CTR) melan-a6 cells. Deleted nucleotides are shown between brackets in red and inserted nucleotides are indicated in green. Bottom: amino acid sequence of the resulting truncated Mgrn1 protein in Mgrn1-KO cells.

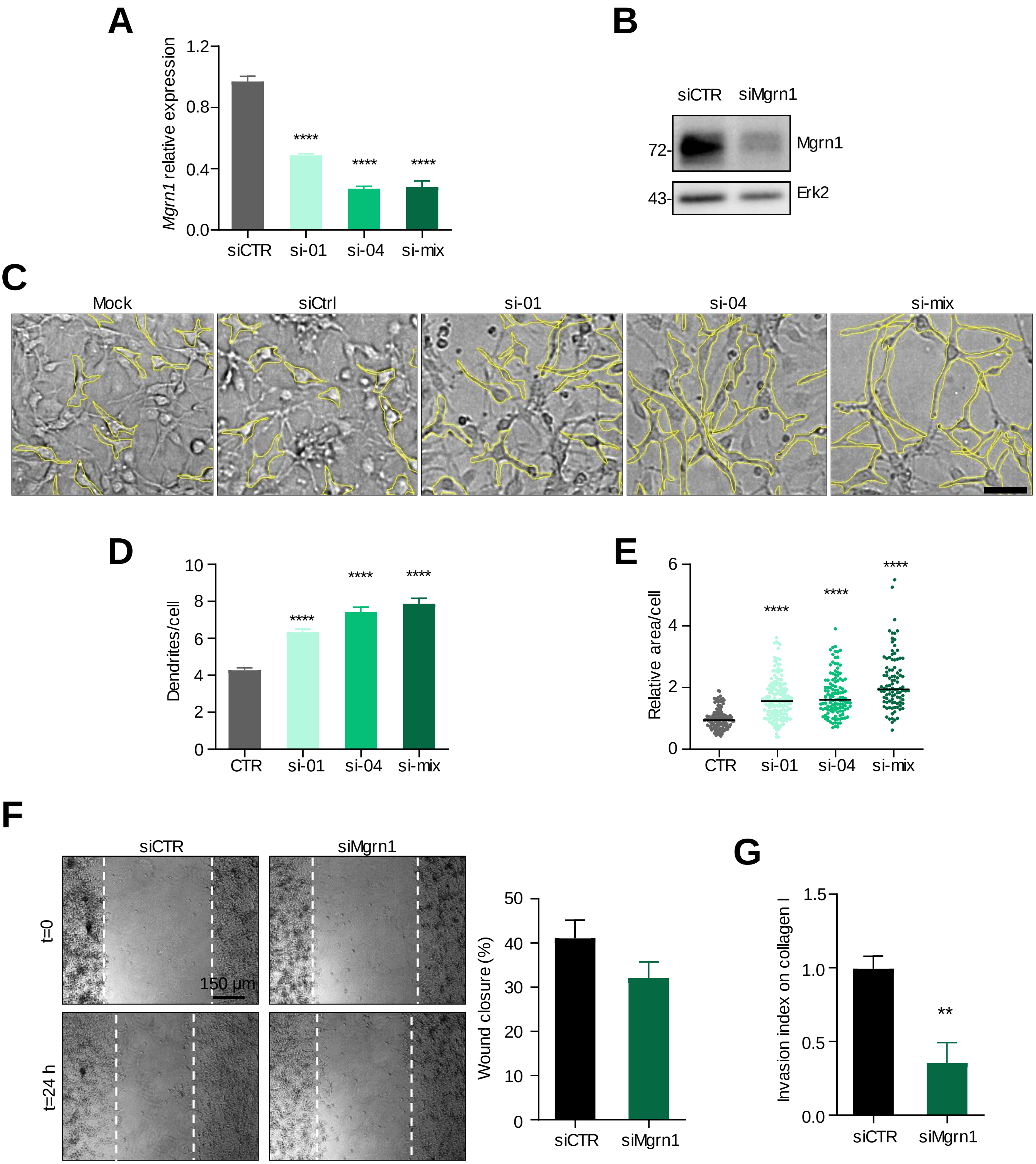

Figure S2. Changes in shape and motility of melan-a6 mouse melanocytes upon downregulation of MGRN1 expression with siRNA.

(A) *Mgrn1* mRNA levels in melan-a6 cells following depletion with siRNA with two different individual siRNAs (si-01 or si-04), or with a stoichiometric mixture of these oligonucleotides (si-mix). siCTR refers to cells treated with a control siRNA. The sequence of all oligonucleotides is specified in Supplemental Table 1.

(B) Representative immunoblot for Mgrn1 in melan-a6 cells after Mgrn1 knockdown with si-mix.

(C) Phase contrast 2D images of Mgrn1-depleted melan-a6 cells. Cells were treated with the indicated siRNA. Their morphology was analyzed after manually drawing around the cell shape using imageJ (yellow).

(D) Quantification of the number of dendrites per cell in melan-a6 cells after Mgrn1 knockdown.

(E) Quantification of the relative area per cell in Mgrn1-depleted melan-a6 cells.

(F) Phase-contrast images of confluent control (siCTR) and Mgrn1-depleted (si-mix Mgrn1) melan-a6 cells migrating to the center of a scratch after 24 h and quantification of the percentage of wound closure.

(G) Invasion index at 50 µm after 24 h for melan-a6 cells treated with control (siCTR) or Mgrn1-directed (siMgrn1) siRNA.

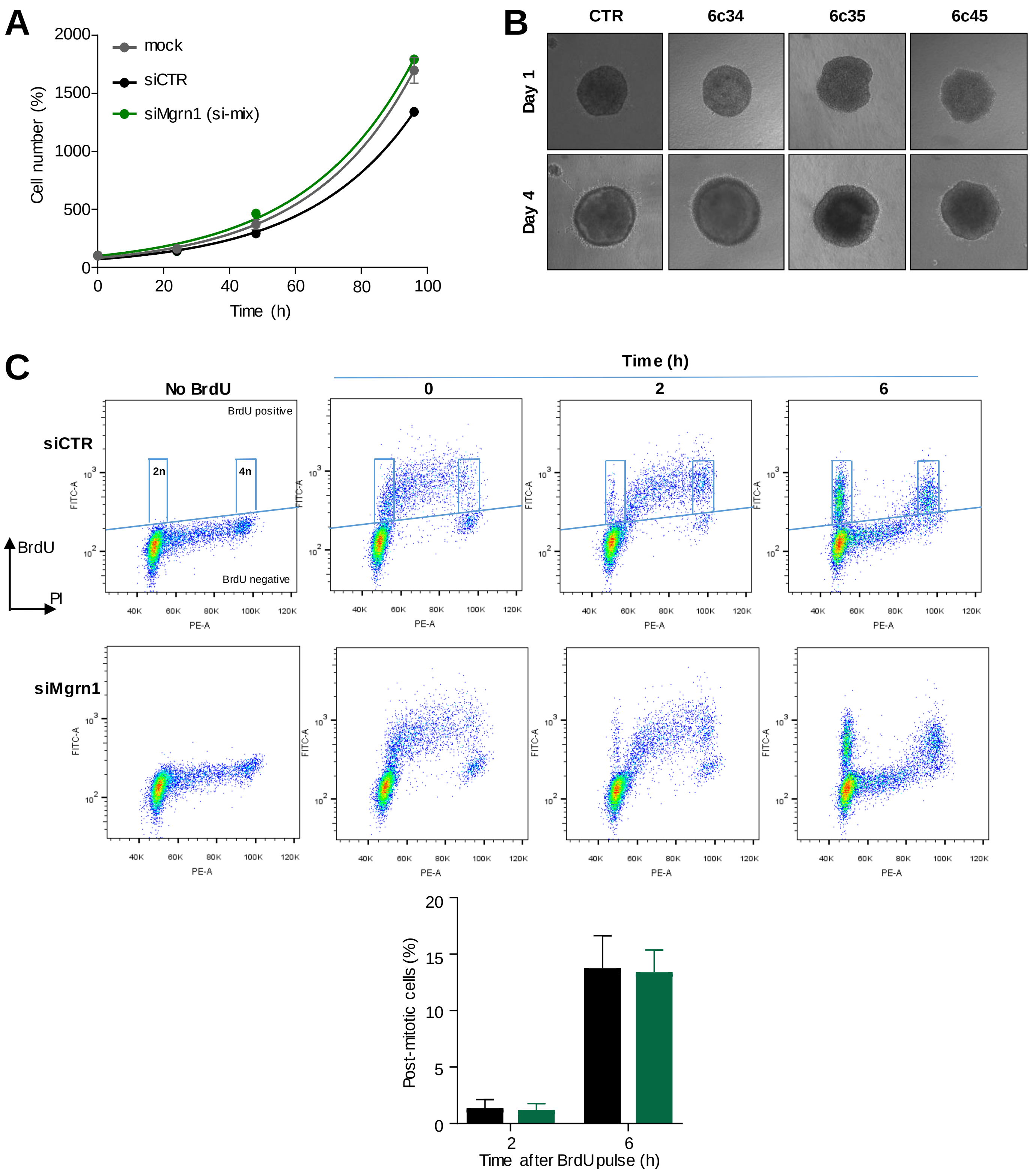

Figure S3. Proliferation rates and cell cycle progression of MGRN1-depleted cells.

(A) Representative images of the growth of spheroids from control (CTR) and *Mgrn1*-KO clones 6c34, 6c35 and 6c45 in a collagen I matrix between days 1 and 4 (upper). The quantification of the increase in area of the spheroids did not yield statistically significant variations.

(B) Growth curves for melan-a6 cells treated with a scrambled control siRNA (siCTR) or with MGRN1-directed siRNA (siMgrn1, si-mix) obtained by cell counting over a period of 4 days. Untreated cells (mock) are also shown for comparison. Upon nonlinear regression, doubling times of roughly 23 h were calculated, with no significant differences for cells treated with control or Mgrn1-directed siRNA.

(C) Cell cycle progression in melan-a6 cells treated with control and Mgrn1-specific siRNA. Cells pulsed with BrdU were chased for the times shown in BrdU-free medium, stained for newly synthesized DNA with an anti-BrdU antibody and for total DNA with propidium iodide (PI), and analyzed in a FACScanto flow cytometer. The histogram shows the percentage of post-mitotic cells, identified as BrdU-positive cells with a 2n chromosome number (PI staining), according to the gating scheme shown for siCTR cells (left column).

A

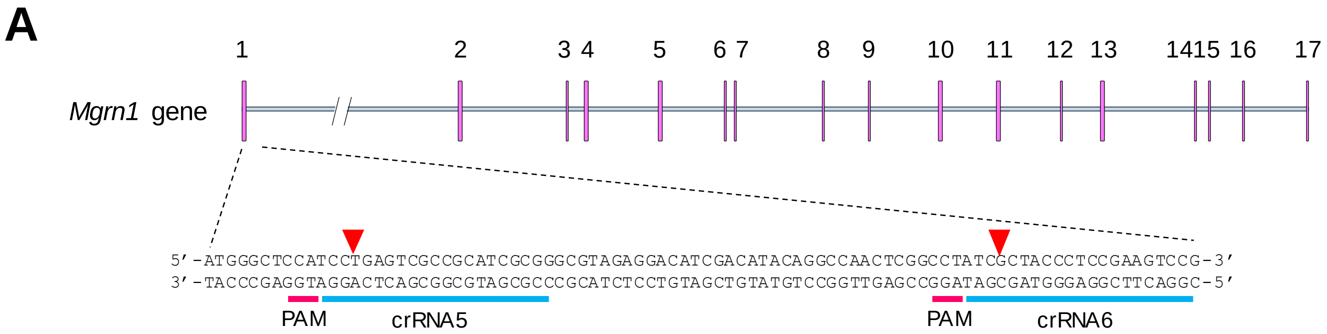

B

crRNA clones	Edited sequences on *Mgrn1* exon 1
CTR	Alleles 1 and 2: $^{-35}$C CTC TCC GGC TGG CCG GTA GAA ACG CGC ACC $^{+1}$ATG GGC TCC ATC **CT**G AGT^{+18}
5c5	Allele 1: $^{-35}$C CTC TCC GGC TGG CCG GTA GAA ACG CGC ACC $^{+1}$ATG GGC TCC ATC CGT GAG T^{+18}<br>Allele 2: $^{-35}$C CT(CTCC GGC TGG CCG GTA GAA ACG CGC ACC) $^{+1}$ATG GG(C) TCC C(A)T CCA GTG AGT^{+18}
CTR	Alleles 1 and 2: ^{58}AAC TCG GCC TAT **CG**C TAC CCT CCG AAG TCC GGA^{90}
6c26	Allele 1: ^{58}AAC TCG G(CC TAT CGC TAC)CCT CCG AAG TCC GGA^{90}<br>Allele 2: ^{58}AAC TCG G(CC TAT CGC TAC CCT CCG)AAG TCC GGA^{90}

```
            1        10        20        30        40        50        60        70        80      532
            |--------+---------+---------+---------+---------+---------+---------+---------| //------|
       CTR  MGSILSRRIAGVEDIDIQANSAYRYPPKSGNYFASHFFMGGEKFDTPHPEGYLFGENMDLNFLGSRPVQFPYVTPAPHEP //------|
  5c5-iso1  MGSIRESPHRGRRGHRHTGQLGLSLPSEVRKLLCFAFFHGRREI
 6c26-iso1  MGSILSRRIAGVEDIDIQANSA----SEVRKLLCFAFFHGRREI
 6c26-iso2  MGSILSRRIAGVEDIDIQANS------EVRKLLCFAFFHGRREI
```

C **D**

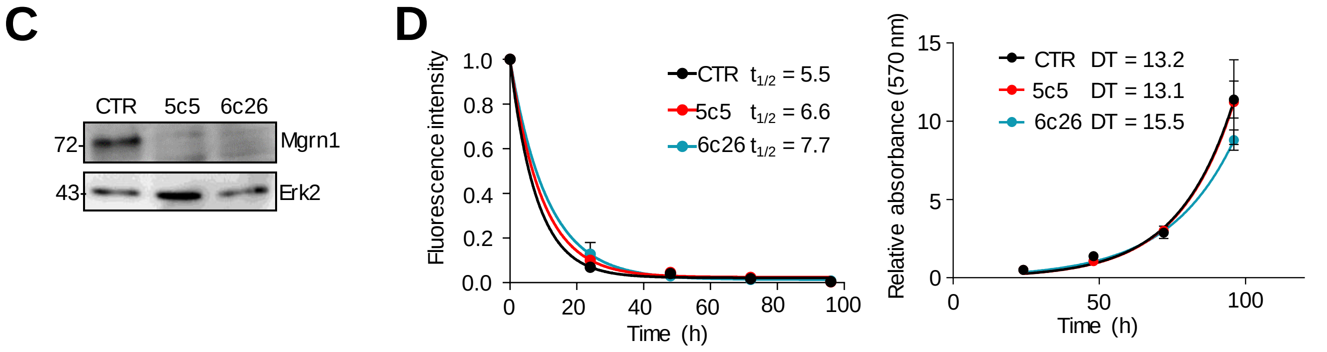

Figure S4. Generation of MGRN1-KO B16 mouse melanoma cells: strategy, validation and effect on the rate of proliferation.

(A) Schematic representation of *Mgrn1* and sequence in exon 1 targeted by crRNA5 and crRNA6. Both 20 nt sequences pair with the DNA target highlighted with a blue bar on the bottom strand, directly upstream of a 5'-NGG adjacent motif (PAM, pink).

(B) Edited sequences (from -35 to +18 nt and from +58 to +90 nt) in exon 1 of the *Mgrn1* gene in Mgrn1-KO clones 5c5 and 6c26, compared with the consensus sequence in control (CTR) B16F10 cells. Deleted nucleotides are represented between brackets in red and inserted nucleotides are indicated in green. The amino acid sequence of the resulting truncated Mgrn1 protein in Mgrn1-KO cells is shown below.

(C) Representative immunoblots for Mgrn1 in Mgrn1-KO B16F10 melanoma clones.

(D) Analysis of cell division of Mgrn1-KO B16F10 cells by measuring the decrease in CFSE fluorescence intensity and calculation of half-lifes (t1/2) (left) and by the MTT assay measuring the increase in relative absorbance at 570 nm and determining the doubling times (DT) (right) for a period of 4 days.

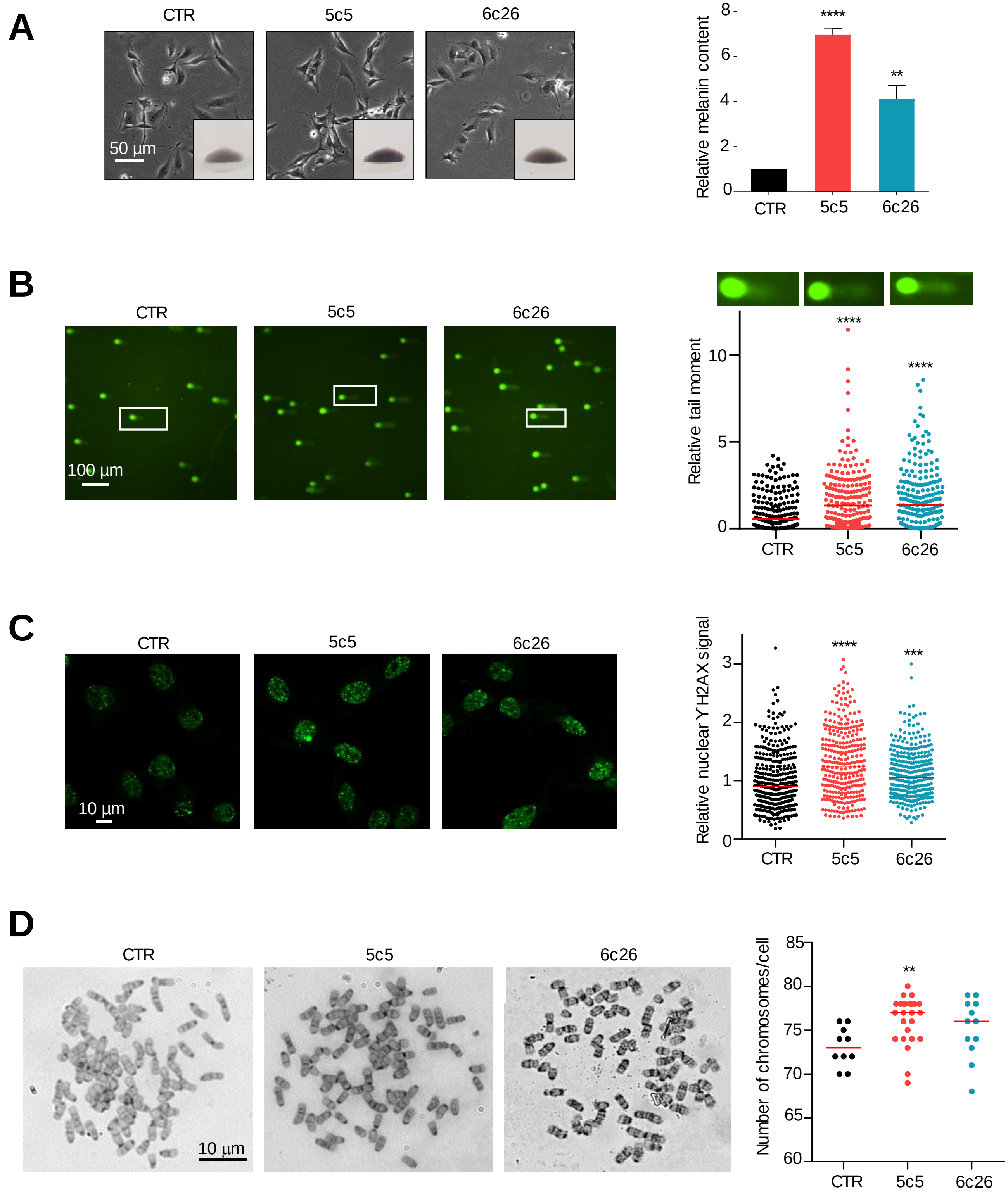

Figure S5. Increased genomic instability in MGRN1-KO B16 mouse melanoma cells.

(A) Micrographs of Mgrn1-null B16F10 cells in 2D cultures. Inserts show representative images of cell pellets. Quantification of the relative melanin content is shown on the right.

(B) Alkaline comet analysis of DNA in control (CTR) and Mgrn1-null B16F10 cells. Histograms show the mean average of the tail moment of Mgrn1-null cells relative to control cells (n = 3 independent experiments, each one with at least 70 comets analyzed). Representative images acquired at 40× magnification are shown above each histogram. ****p < 0.0001

(C) Confocal images of γ-H2AX immunostaining in Mgrn1-KO and control B16F10 cells. Histograms show γ-H2AX nuclear intensity of Mgrn1-null cells relative to controls.

(D) Micrographs of metaphase chromosome spreads of control and Mgrn1-KO B16F10 cells after Giemsa staining. The dot plot represents the number of chromosomes per cell.

Supplementary Table 1. Sequence of control and *Mgrn1*-directed siRNA

Name	Sequence
SiGENOME Non-Targeting siRNA #2 (siCTR)	UAAGGCUAUGAAGAGAUAC
siGENOME Mgrn1-1 (si-01)	GAUGGCAGCUUCUCCGUGA
siGENOME Mgrn1-2 (si-02)	GAGAUGAGCCGUCCCUAAA
siGENOME Mgrn1-3 (si-03)	GCACUCUAGUUCUGACAGU
siGENOME Mgrn1-4 (si-04)	CGUAGAGGACAUCGACAUA

All oligonucleotides were from Dharmacon. In most cases, si-01, si-04 and/or a stoichiometric mix of these oligonucleotides (si-mix) were employed, at a final total oligonucleotide concentration of 30 nM.

Supplementary Table 2. Details of crispr-RNAs for knockout of *Mgrn1* expression in mouse melanocytic cells.

crispr-RNA	Target sequence	PAM	Genomic Location (strand)	Score efficiency	Off-targets
cr-RNA5	CGCGATGCGGCGACTCAGGA	TGG	Chr16: 4886393-4886415 (-)	95.1	11
cr-RNA6	GGACTTCGGAGGGTAGCGAT	AGG	Chr16: 4886450-4886472 (-)	92.6	37

Target and protospacer adjacent motif (PAM) sequences, genomic location, score efficiency and number of off-targets for each cr-RNA.

Supplementary Table 3. Antibodies used.

Target	Supplier	Catalog number	Working Dilution
Actin	Sigma-Aldrich, USA	A2066	1:5000[a]
γ-H2AX	Abcam, UK	ab2893	1:100[b]
MGRN1	Proteintech, UK	11285-1-AP	1:2000[a]
MITF	Cell Signaling USA	12590	1:5000[a]
mKi67	Abcam, UK	ab16667	1:100[c]
HA	Sigma-Aldrich, USA	H9658	1:2000[b]
8-oxodG	Trevigen, USA	4354-MC-050	1:250[b]

The antibodies specified were employed for [a] Western blot, [b] confocal microscopy or [c], immunohistochemistry

Figure 1, panel E

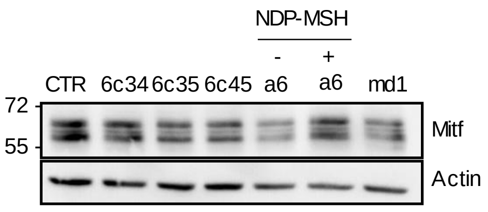

Uncropped, original western-blots:

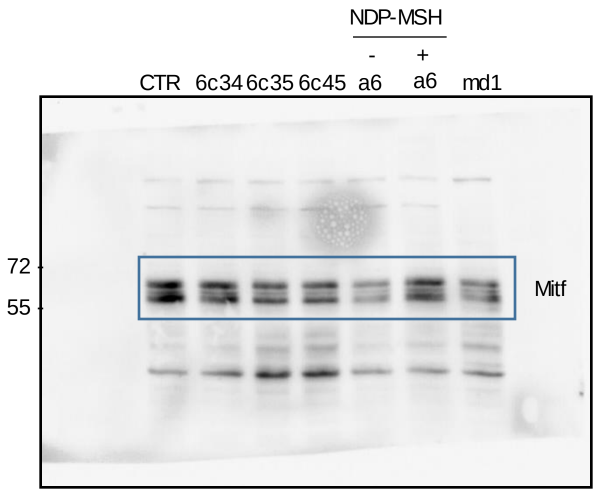

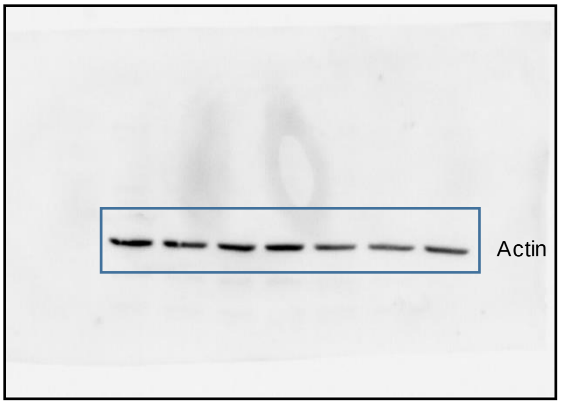

Figure 3, panel D

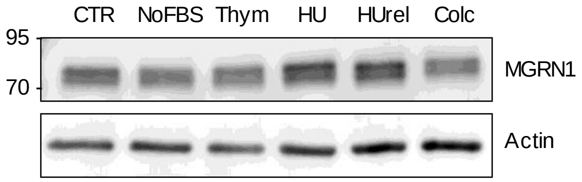

Uncropped, original western-blots:

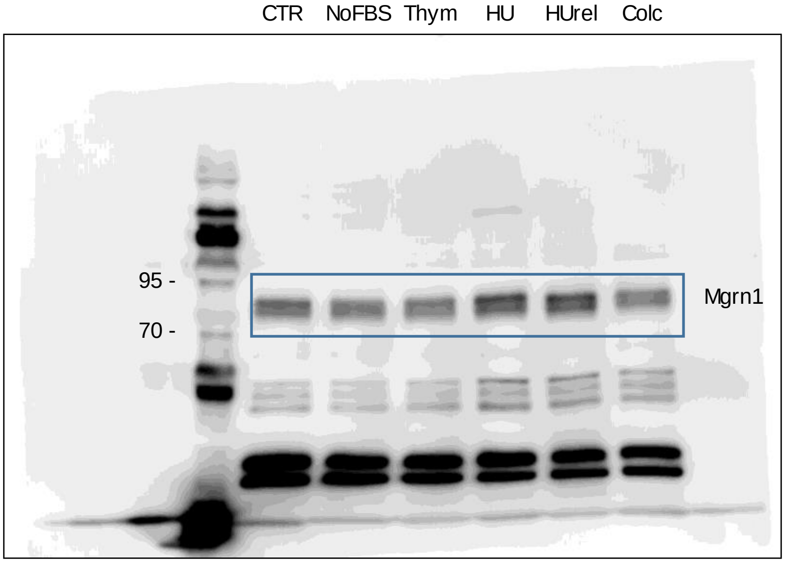

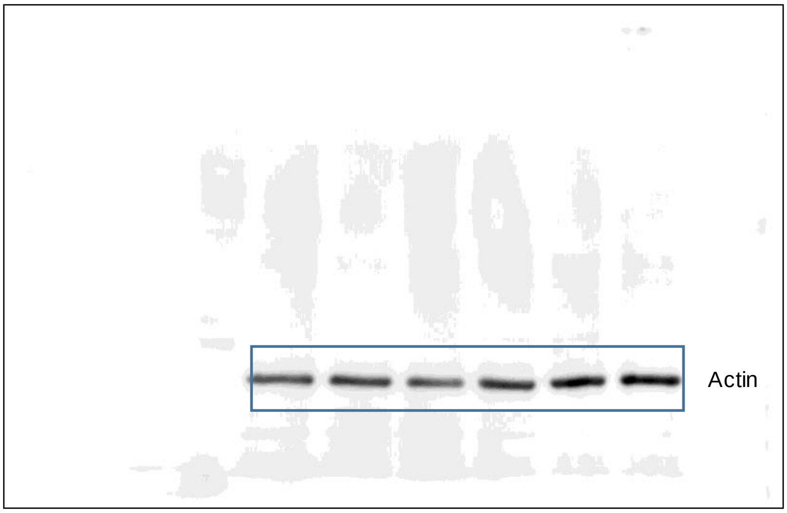

Figure 3, panel E

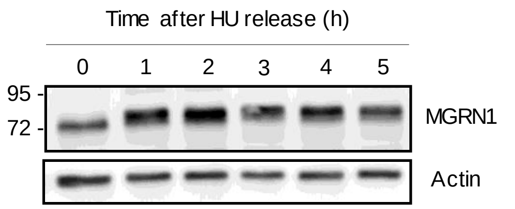

Uncropped, original western-blots:

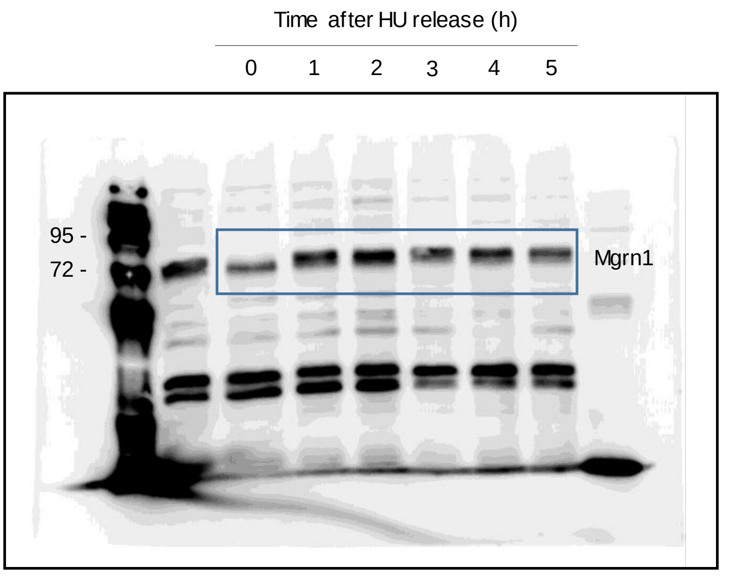

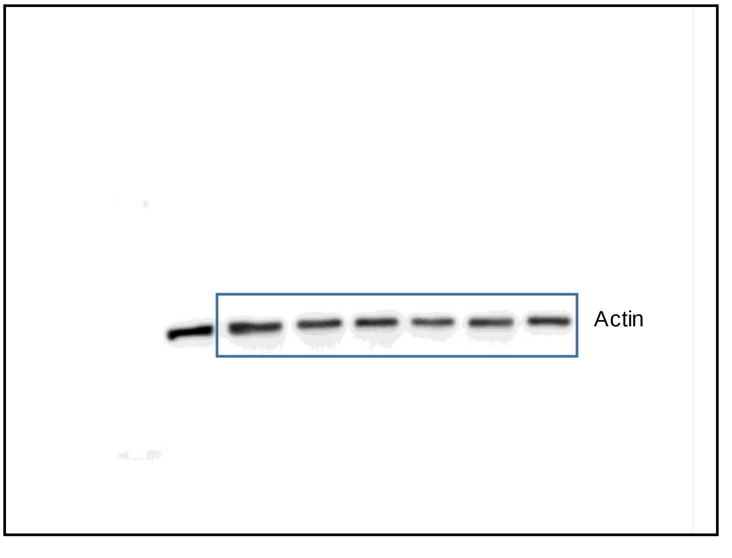

Supp Figure 1, panel B

Immunoblots

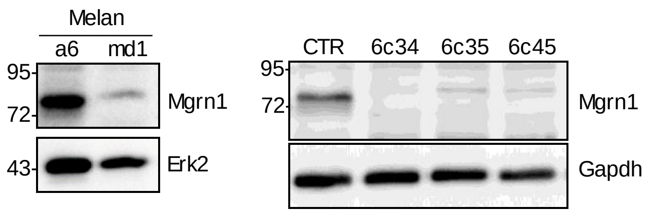

Uncropped, original western-blots:

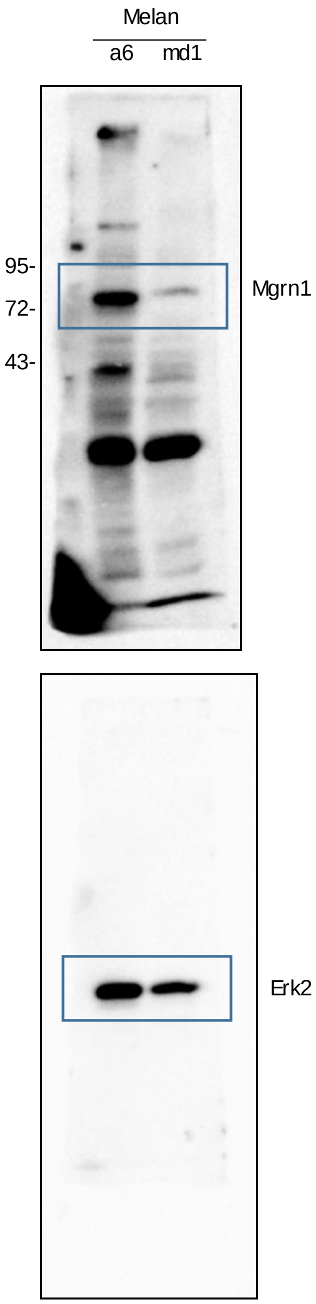

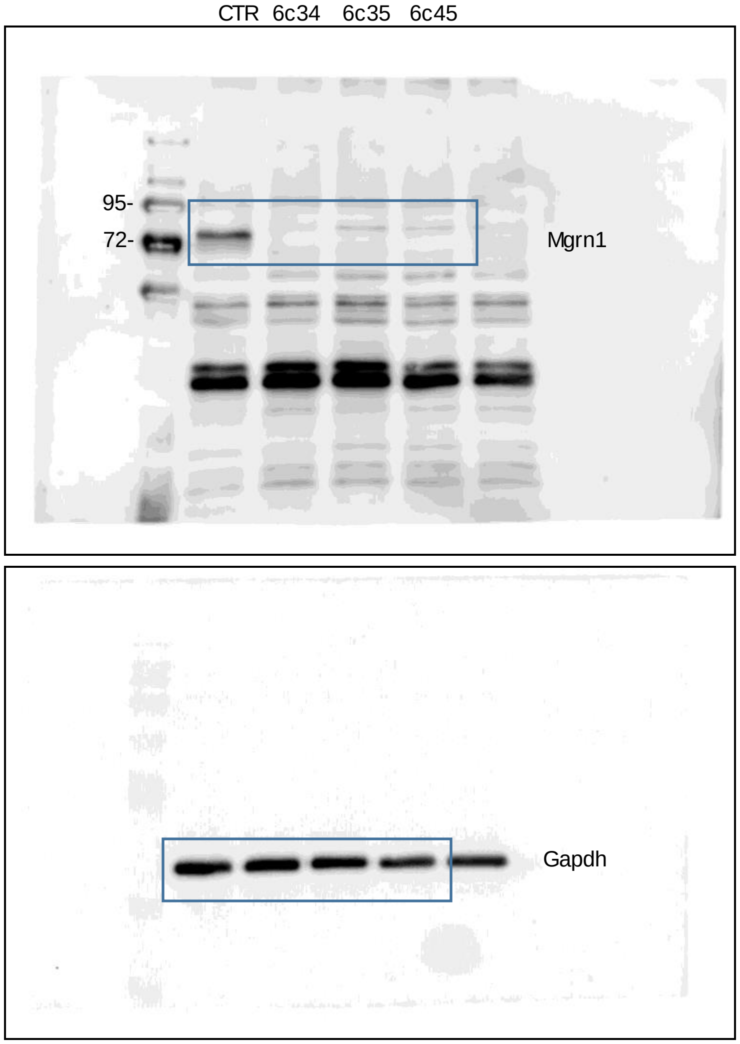

Supp Figure 2, panel B

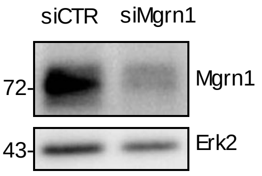

Uncropped, original western-blots:

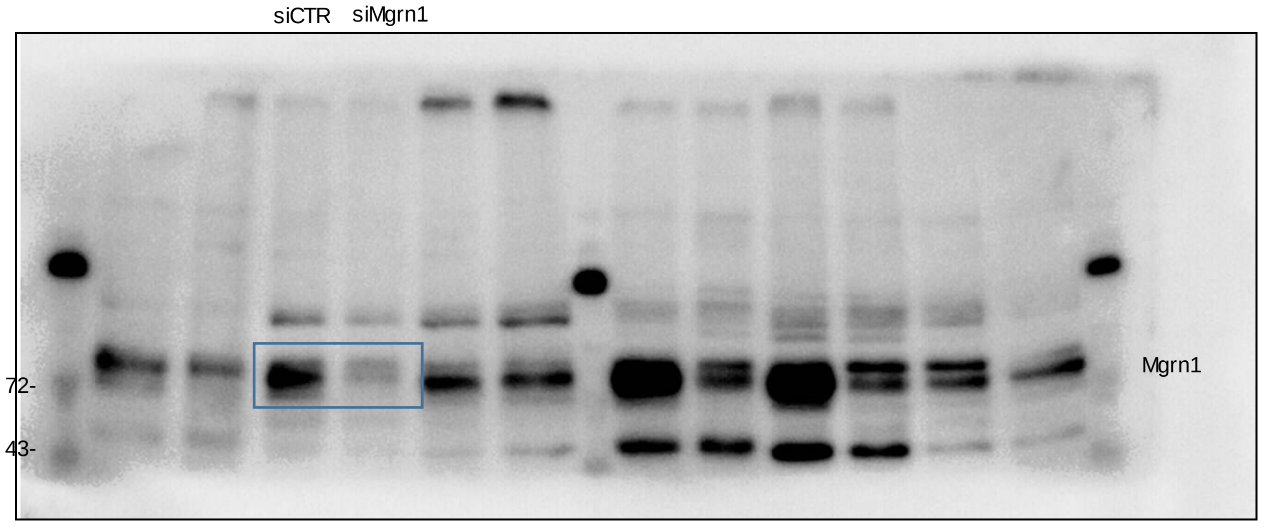

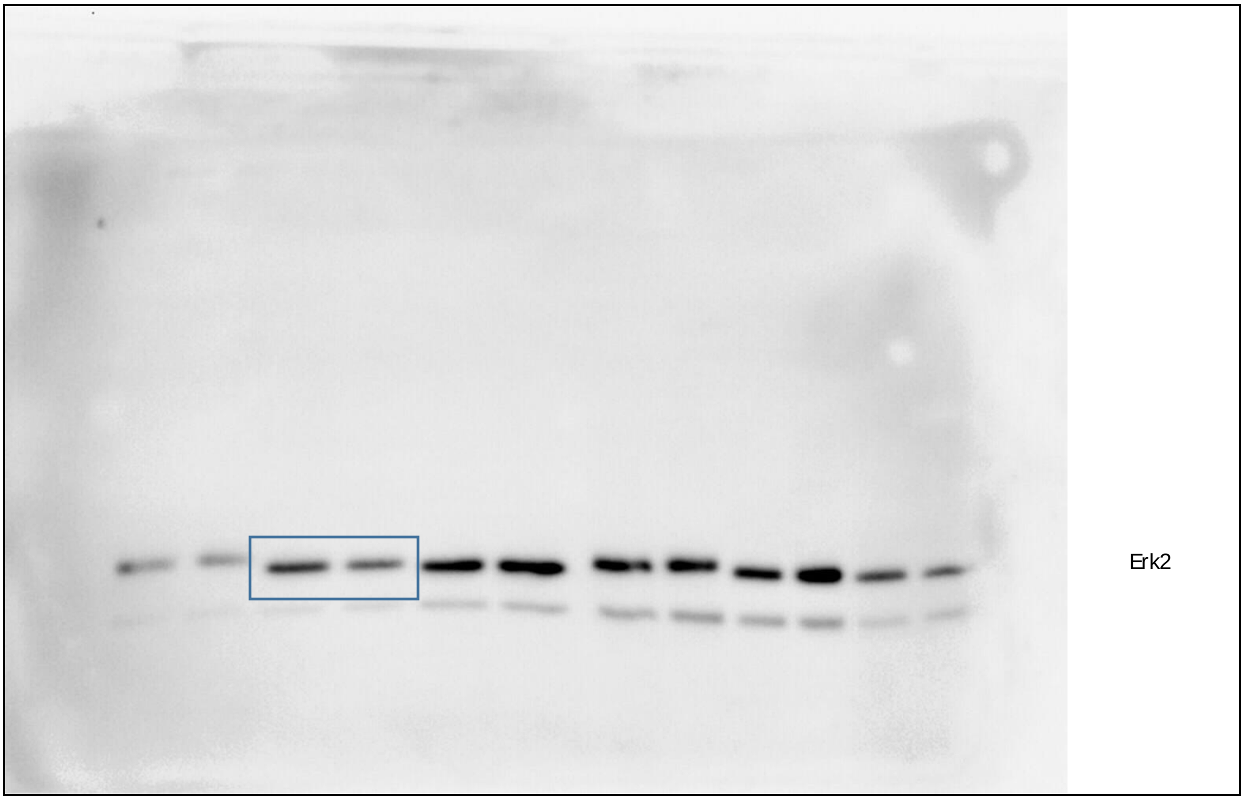

Supp Figure 4, panel C

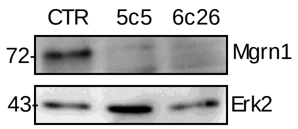

Uncropped, original western-blots:

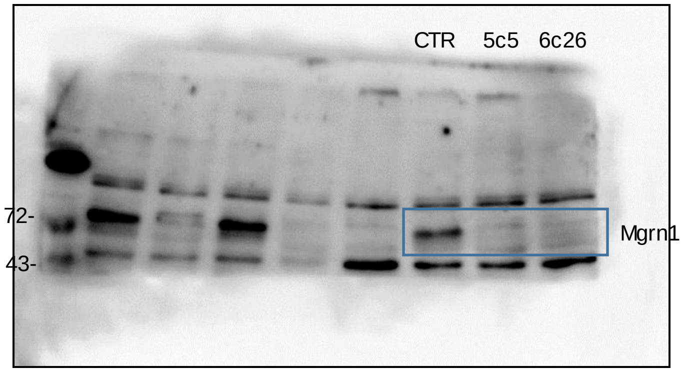

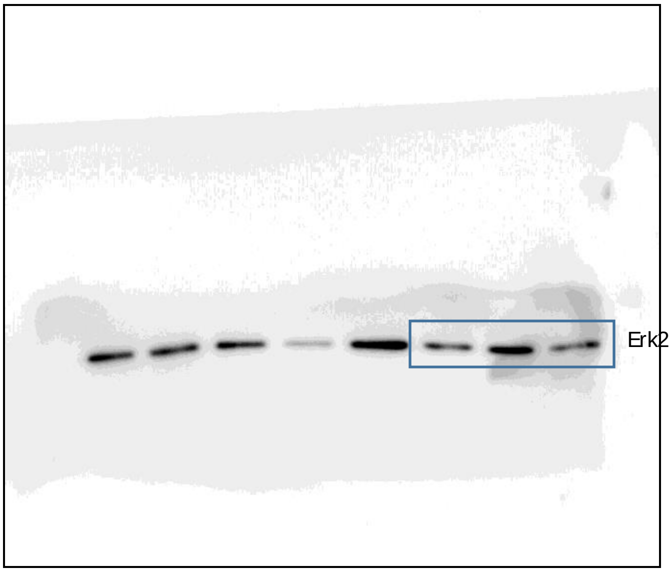

Supplement: Supplementary file 1 [file cancers-12-02840-s001.pdf]
